# IL-33/ST2 signaling in ILC2s drives exhaustion and myeloid skewing of HSCs in response to hematopoietic stress and aging

**DOI:** 10.1016/j.isci.2025.112378

**Published:** 2025-04-08

**Authors:** Pascal Naef, Carla A. Jaeger-Ruckstuhl, Noah Schnüriger, Stefan Forster, Inês Monteiro, Daniel Brigger, Alexander Eggel, Kai Kessenbrock, Carsten Riether, Adrian F. Ochsenbein

**Affiliations:** 1 Department for BioMedical Research (DBMR), University of Bern, 3008 Bern, Switzerland; 2 Department of Medical Oncology, Inselspital, Bern University Hospital, University of Bern, 3010 Bern, Switzerland; 3 Graduate School of Cellular and Biomedical Sciences, University of Bern, 3012 Bern, Switzerland; 4 Department of Rheumatology and Immunology, Inselspital, Bern University Hospital, 3008 Bern, Switzerland; 5 Department of Biological Chemistry, University of California, Irvine, Irvine, CA 92697, USA

**Keywords:** Immune response, Cell biology

## Abstract

Inflammatory cues affect hematopoietic stem cell (HSC) homeostasis and drive proliferation and myeloid skewing of HSCs. The HSC niche in the bone marrow (BM) is populated by a variety of stromal and immune cells that sense and respond to cellular stress. We investigated how BM-resident type 2 innate lymphoid cells (ILC2s) regulate HSC homeostasis and differentiation in steady state, during aging, and after genotoxic stress. We documented that PDGFR-α^+^sca-1^+^ mesenchymal stromal cells in the BM produced interleukin (IL)-33 with elevated levels after irradiation and during aging. IL-33/ST2 signaling in BM-resident ILC2s activated MAPK/NF-κB/JAK-STAT signaling and induced cytokine secretion. IL-6 and granulocyte-macrophage colony-stimulating factor (GM-CSF), secreted by ILC2s, promoted HSCs to proliferate and differentiate into the myeloid lineage. Taken together, we identified that IL-33 produced by MSCs induced ILC2s to secrete myeloid differentiation factors leading to myeloid-skewed HSCs with reduced self-renewal during aging.

## Highlights

• IL-6 and GM-CSF secreted by ILC2s promote proliferation and differentiation of HSCs
• Irradiation and aging induce elevated IL-33 levels and ILC2 activation
• Activated ILC2s induce the expansion of functionally impaired and myeloid skewed HSCs

## Graphical abstract

**Figure:**
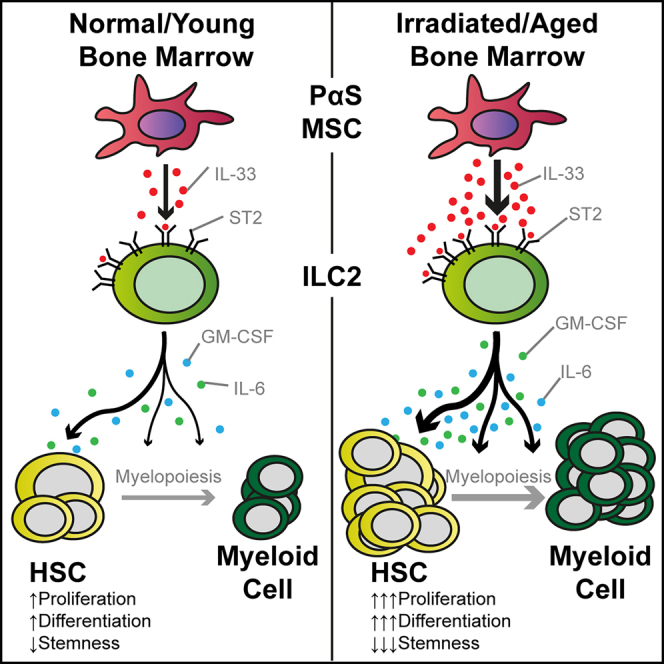

## Introduction

Hematopoietic stem cells (HSCs) are pluripotent cells with the capacity to differentiate into all mature blood cells. They are at the top of the hematopoietic hierarchy and have a unique property for self-renewal.^1^^,^^2^ Adult HSCs reside at specific sites in the bone marrow (BM), the so-called HSC niche.^3^^,^^4^^,^^5^^,^^6^^,^^7^ The HSC niche consists of soluble and cellular components, for example, various immune cells which influence the cell fate of HSCs by regulating their self-renewal, proliferation, and differentiation capacities.^1^^,^^8^^,^^9^ Although not classically considered niche cells, immune cells are an integral part of the BM microenvironment and contribute to the regulation of hematopoiesis. For example, CD4^+^CD25^+^ regulatory T cells, Th2-skewed CD4^+^ helper cells, and cytotoxic T cells have been reported to regulate HSCs' proliferation and differentiation, mainly by secreting effector cytokines, including interleukin (IL)-3 and granulocyte-macrophage colony-stimulating factor (GM-CSF).^10^^,^^11^^,^^12^

Type 2 innate lymphoid cells (ILC2s) are tissue-resident cells that have been identified in different lymphoid and non-lymphoid organs, e.g., in the intestine, lung, and BM.^13^ ILC2s play an essential role as regulators of tissue repair, parasite clearance, and airway inflammation.^14^^,^^15^^,^^16^^,^^17^^,^^18^^,^^19^^,^^20^^,^^21^ Like T and B cells, ILC2s originate from common lymphoid progenitors in the BM and express the receptors for IL-2 and IL-7. However, they are not directly activated by antigen-specific signaling, but they are triggered by various cytokines, such as IL-25, IL-33, and thymic stromal lymphopoietin (TSLP), and produce a variety of effector cytokines, including IL-2, IL-5, IL-6, IL-9, IL-13, GM-CSF, and the epidermal growth factor-related protein amphiregulin (AREG).^22^^,^^23^^,^^24^ Although many of these cytokines are known to regulate hematopoietic stem and progenitor cells (HSPCs), it remains incompletely understood how ILC2s contribute to hematopoiesis. While their role in supporting hematopoietic recovery after treatment with 5-fluorouracil through the secretion of GM-CSF has recently been reported^25^ their contribution to hematopoiesis at steady-state and aging is currently not known.

A crucial regulator of ILC2 activity is IL-33, an IL-1 family member cytokine involved in type 2 immunity, allergy, and inflammation.^26^^,^^27^^,^^28^ Various types of cells, including endothelial, epithelial, and stromal cells, express IL-33 in the nucleus.^24^^,^^29^^,^^30^^,^^31^ Intranuclear IL-33 is associated with chromatin and functions as a transcriptional repressor.^32^^,^^33^ Besides its intranuclear location, IL-33 can be released upon mechanical stress or necrosis and functions as an alarmin.^34^^,^^35^^,^^36^ Released IL-33 signals through a heterodimeric receptor complex composed of ST2 and the IL-1 receptor accessory protein (IL1R-AcP).^26^^,^^37^ The binding of IL-33 to its receptor induces MyD88 recruitment, activates the nuclear factor kappa B (NF-κB) and MAPK pathways, and induces target gene expression. The IL-33 receptor ST2 is expressed on various adaptive and innate immune cells, including ILC2s.^17^^,^^18^^,^^38^^,^^39^^,^^40^^,^^41^^,^^42^^,^^43^^,^^44^^,^^45^^,^^46^

In the present study, we analyzed the role of IL-33/ST2 signaling in BM-resident ILC2s and their influence on hematopoiesis in steady-state, after genotoxic stress, and during aging. We found a basal production of IL-33 by BM-derived stromal cells with increased levels after irradiation and during aging. IL-33/ST2 signaling in BM-resident ILC2s activated NF-κB, MAPK, and JAK-STAT pathways and induced the secretion of cytokines. IL-6 and GM-CSF produced by ILC2s promoted the proliferation and myeloid differentiation of quiescent HSPCs. Increased IL-33 levels after irradiation induced the expansion of HSPCs with reduced self-renewal capacity. Similarly, aging resulted in increased levels of IL-33/ST2 signaling in ILC2s, resulting in the expansion of functionally impaired and myeloid-skewed HSPCs. Thus, IL-33-stimulated ILC2s regulate myelopoiesis and crucially contribute to the aging of HSPCs.

## Results

### Absence of ILC2s or IL-33/ST2 signaling ILC2s reduces myelopoiesis

**Figure 1:**
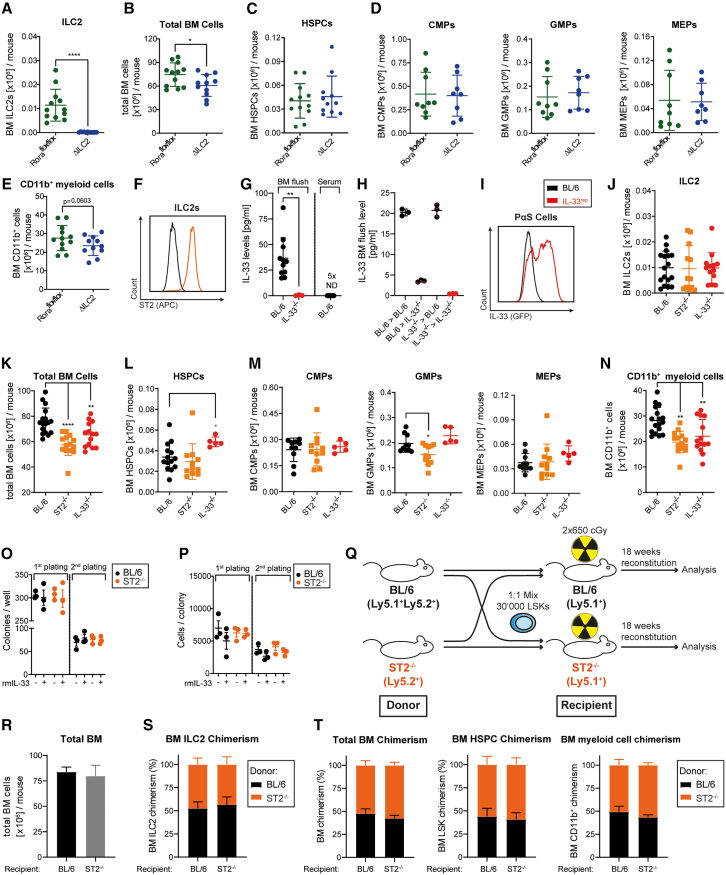
IL-33/ST2 signaling in ILC2s promotes myelopoiesis (A) BM ILC2 numbers in Rora^flox/flox^ (*n* = 12) and Rora^flox/flox^ x IL7RCre (ΔILC2, *n* = 11) mice (pooled data from 3 independent experiments). (B) Total BM cell numbers in Rora^flox/flox^ and ΔILC2 mice. (C–E) Total BM hematopoietic stem and progenitor cell (HSPC, C), common myeloid progenitor (CMP), granulocyte-macrophage progenitor (GMP), megakaryocyte-erythroid progenitor (MEP, D), and myeloid cell (CD11b+, E) numbers in Rora^flox/flox^ and ΔILC2 mice. (F) Representative histogram of the ST2 expression on ILC2s, measured by flow cytometry; black: ST2^−/−^mouse; orange: BL/6. (G) IL-33 BM fluid levels in BL/6 (*n* = 11) and IL-33^−/−^ (*n* = 3) mice, and IL-33 serum levels in BL/6 mice (*n* = 5) measured with a U-Plex multiplex assay from MSD (pooled data from 3 independent experiments). (H) Results of the BL/6-IL-33^−/−^ chimera experiment. 10 weeks post-transplantation, recipients were sacrificed, and the BM fluid was analyzed for IL-33. (I) Representative histogram of the IL-33 expression on PDGFR-α^+^sca-1^+^ mesenchymal stromal cells (PαS cells), measured in BL/6 (black line) and IL-33-GFP-reporter mice (IL-33^rep^; red line) by flow cytometry. (J) ILC2 numbers in BL/6 (*n* = 17), ST2^−/−^ (*n* = 14), and IL-33-knockout (IL-33^−/−^, *n* = 14) mice (pooled data from 6 independent experiments). (K–N) Total BM cell (K), HSPC (L), CMP, GMP, MEP (M), and myeloid cell (N) numbers in BL/6 (*n* = 13–17), ST2^−/−^ (*n* = 14) and IL-33^−/−^ (*n* = 9–14) mice. (O and P) Colony forming assay performed with fluorescence-activated cell sorting (FACS)-purified HSPCs from naive BL/6 or ST2^−/−^ mice, in the presence or absence of recombinant murine (rm)IL-33 (100 ng/ml). The first plating was performed with 1 × 10^3^ primary HSPCs. The colonies were counted (O), and the number of cells per colony was evaluated (P) 7 days post-plating. 1 × 10^4^ cells were replated in methylcellulose with or without rmIL-33 (100 ng/ml). The replating was analyzed 7 days post-plating (*n* = 3 per condition). (Q–T) BL/6 vs. ST2^−/−^ competitive chimera experiment. (Q) Experimental setup for the competitive chimera experiment: 1.5 × 10^4^ Ly5.1 ^+^ 5.2^+^ BL/6 and 1.5 × 10^4^ Ly5.2+ ST2^−/−^ HSPCs were co-transplanted into lethally irradiated (2 × 60cGy) Ly5.1^+^ BL/6 (*n* = 4, males) or ST2^−/−^ (*n* = 5, males) recipients. The chimerism was assessed 18 weeks post-transplantation. (R) Total BM cell numbers of BL/6 and the ST2^−/−^ recipients 18 weeks post-transplantation. (S and T) Percent distribution (chimerism) of ILC2 (S), total BM cells, HSPC, and myeloid cells (T) 18 weeks post-transplantation. Data are represented as mean ± SD. Statistics: two-tailed Student’s t test (A, B, and E), one-way ANOVA (I–L); ∗*p* < 0.05, ∗∗*p* < 0.01, ∗∗∗*p* < 0.001, ∗∗∗∗*p* < 0.0001. See also Figure S1.

To investigate the role of BM-resident ILC2s in steady-state hematopoiesis, we compared the BM-cellularity and composition of naive Rora^flox/flox^ x IL7RCre mice, which cannot develop ILC2s (ΔILC2 mice) and Rora^flox/flox^ control animals.^47^ BM-resident ILC2s were defined as lin^−^sca-1^+^CD90.2^+^CD25^+^CD127^+^ cells (Figure S1A). As expected, ΔILC2 mice were devoid of BM-resident ILC2s, while Rora^flox/flox^ control mice had normal ILC2 numbers (Figures 1A and S1B). ΔILC2 mice had a reduced BM cellularity compared to Rora^flox/flox^ control mice (Figure 1B). This difference was due to reduced numbers of CD11b^+^ myeloid cells, while the counts of hematopoietic stem and progenitor cells (HSPCs; defined as lin^−^sca-1^+^ckit^+^), common myeloid progenitors (CMPs; defined as lin^−^sca-1^−^ckit^+^FcγR^−^CD34^+^), granulocyte-monocyte progenitors (GMPs; defined as lin^-^sca-1^−^ckit^+^FcγR^+^CD34^+^), and megakaryocyte-erythroid progenitors (MEPs; defined as lin^-^sca-1^−^ckit^+^FcγR^−^CD34^−^) were comparable between ΔILC2 and Rora^flox/flox^ control mice (Figures 1C–1E and S1C–S1F). This suggests that ILC2s regulate myelopoiesis during homeostasis.

ST2, the receptor for IL-33, is present on various cells in the BM niche. While HSPCs, GMPs, and myeloid cells were ST2-negative, BM-resident ILC2s strongly expressed ST2 (Figures 1F, S1G, and S1H). CMPs also expressed ST2 but at lower levels. ILC2s were the only ILC subpopulation in the BM that expressed ST2 (Figures S1I and S1J). Interestingly, IL-33 was detectable in the BM fluid but not in the sera of BL/6 mice, suggesting a local source of IL-33 production in the BM (Figure 1G). To investigate whether IL-33 is released by hematopoietic cells or by radioresistant stromal cells, we generated chimeric mice by injecting BL/6 or IL-33^−/−^ BM cells into either lethally irradiated BL/6 or IL-33^−/−^ recipients and measured the BM IL-33 concentration in the BM fluid 10 weeks post-transplantation (Figure S1K). Our results indicated that IL-33 was mainly produced by irradiation-resistant stromal cells in the BM (Figure 1H). This finding was confirmed in an IL-33-GFP reporter mouse, where we identified PDGFR-α+sca-1+ mesenchymal stromal cells (PαS) as a main source of IL-33 production, whereas neither CD31^+^ endothelial nor EpCam^+^ epithelial cells expressed IL-33^48^ (Figures 1I and S1L–S1M).

Next, we compared the BM-cellularity and composition of naive BL/6, ST2-knockout (ST2^−/−^), and IL-33-knockout (IL-33^−/−^) mice. ILC2 numbers in the BM of all 3 mouse strains were comparable, indicating that IL-33/ST2 signaling did not affect the development and maintenance of BM-resident ILC2 (Figure 1J). However, like ΔILC2 mice, ST2^−/−^ and IL-33^−/−^ mice had a significantly reduced BM cellularity compared to BL/6 control animals (Figure 1K). IL-33- or ST2-deficiency did not influence the numbers of HSPCs, CMPs, GMPs, and MEPs, except for a slight increase in HSPCs in IL-33^−/−^ mice and a reduction of GMPs in ST2^−/−^ mice (Figures 1L–1M). The increase in the HSPC compartment in IL-33^−/−^ mice was due to elevated numbers of long-term hematopoietic stem cells (LT-HSCs; defined as lin^−^sca-1^+^ckit^+^CD150^+^CD34^−^) and type 2 multipotent progenitors (MPP2s; defined as lin^-^sca-1^+^ckit^+^CD48^+^CD34^+^) (Figure S1N). Consistent with what we have observed in ΔILC2 mice, the number of myeloid cells decreased in ST2- and IL-33-deficient mice compared to BL/6 controls (Figure 1N). Based on these data, we hypothesized that IL-33/ST2 signaling in BM-resident ILC2s regulates steady-state myelopoiesis.

To analyze a potential direct effect of IL-33/ST2 signaling on HSPCs, we plated HSPCs in methylcellulose with or without recombinant mouse (rm)IL-33 and assessed the colony-forming capacity. The addition of rmIL-33 did not affect the number or size of colonies formed by BL/6 and ST2^−/−^ HSPCs, as shown in two consecutive platings (Figures 1O–1P). To confirm this finding *in vivo*, we performed a competitive repopulation experiment by co-injecting Ly5.1^+^Ly5.2^+^ HSPCs and Ly5.2^+^ ST2^−/−^ HSPCs at a 1:1 ratio into lethally irradiated Ly5.1^+^ BL/6 or ST2^−/−^ recipients (Figure 1Q). ST2-proficient and ST2-deficient HSPCs similarly reconstituted recipient mice as indicated by similar numbers of total BM cells (Figure 1R) and a 50:50% chimerism in HSPCs and myeloid cells (Figures 1S and 1T). These findings exclude a potential direct effect of IL-33/ST2 signaling on HSPCs or an indirect impact via radio-resistant stromal cells.

### Transfer of ST2-competent ILC2s into ST2- or ILC2-deficient recipients normalizes myelopoiesis and bone marrow cellularity

**Figure 2:**
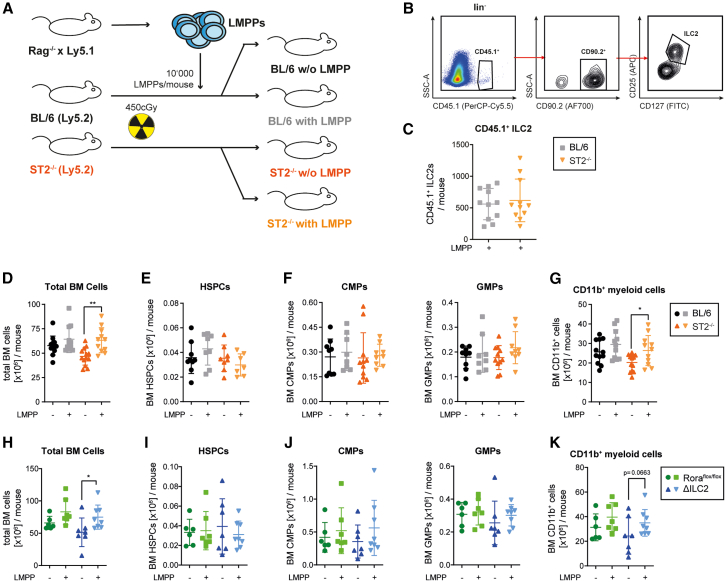
Transfer of ST2-competent ILC2s into ST2^−/−^ recipients normalizes myelopoiesis (A) Experimental layout of the LMPP transfer experiment. Lymphoid-primed multipotent progenitor cells (lin^−^sca-1^+^cKit^+^CD135^+^ LMPPs) were FACS-sorted from Rag1^−/−^ x CD45.1 donors and then transplanted into sub-lethally (450 cGy) irradiated BL/6 and ST2^−/−^ recipients. 18 weeks post-transplantation, the BM compartment was analyzed. (B) FACS-gating strategy for BM-resident CD45.1^+^ ILC2s. (C) Ly5.1^+^ ILC2 numbers in the BM of Ly5.2^+^ BL/6 (*n* = 11) and ST2^−/−^ (*n* = 11) mice. (D) Total BM cell numbers in BL/6 (*n* = 11) and ST2^−/−^ (*n* = 13) LMPP-recipients, and BL/6 (*n* = 12) and ST2^−/−^ (*n* = 13) control mice 18 weeks post-transplantation (pooled data from 4 independent experiments). (E–G) BM CD45.2^+^ HSPC (E), CMP, GMP (F), and myeloid cell numbers (G) in BL/6 and ST2^−/−^ LMPP-recipients and control mice 18 weeks post-transplantation. (H–K) Rora^flox/flox^ and ΔILC2 mice were used as recipients of Rag1^−/−^ x CD45.1 LMPPs in a similar experimental setup (pooled data from 2 independent experiments). (H) Total BM cell numbers in Rora^flox/flox^ (*n* = 7) and ΔILC2 (*n* = 8) LMPP-recipients and Rora^flox/flox^ (*n* = 6) and ΔILC2 (*n* = 7) control mice 18 weeks post-transplantation. (I-K) BM CD45.2^+^ HSPC (I), CMP, GMP (J), and myeloid cell (K) numbers in Rora^flox/flox^ and ΔILC2 LMPP-recipients and control mice 18 weeks post-transplantation. Data are represented as mean ± SD. Statistics: two-tailed Student’s t test; ∗*p* < 0.05, ∗∗*p* < 0.01. See also Figure S2.

Since IL-33- and ST2-deficient, as well as ΔILC2 mice, had reduced myeloid cell numbers compared to control animals, we hypothesized that ILC2s are crucial to maintain myelopoiesis in steady state. To test this hypothesis, we transplanted Rag1^−/−^ x Ly5.1^+^ lymphoid-primed multipotent progenitors (LMPPs) into sub-lethally irradiated BL/6 or ST2^−/−^ recipients (Figures 2A and S2A).^49^ Rag1^−/−^ LMPPs have the potential to differentiate into innate lymphoid cells, including ILC2s, but not into T or B cells. Indeed, Rag1^−/−^ LMPPs gave rise to ILC2s as detected in the BM 18 weeks post-transplantation (Figures 2B and 2C). In contrast, no CD11b^+^ myeloid cells or myeloid progenitors originated from Ly5.1 LMPPs (Figures S2B–S2E). The LMPP transfer rescued the deficient BM-cellularity of ST2^−/−^ mice, while it did not influence the BM cell counts of BL/6 control animals (Figure 2D). HSPC numbers in the BM were not affected upon LMPP transfer in BL/6 and ST2^−/−^ mice, while the number of Ly5.2^+^ myeloid cells was significantly increased in ST2^−/−^ mice after LMPP transfer (Figures 2E–2G).

Next, we transferred LMPPs from Rag1^−/−^ x Ly5.1^+^ mice into ΔILC2 and Rora^flox/flox^ mice. The LMPP transfer increased the number of total BM cells and CD11b^+^ myeloid cells in ΔILC2, but not Rora^flox/flox^ mice, while HSPC numbers remained unaffected (Figures 2H–2K). Thus, IL-33/ST2 signaling in ILC2s promotes myelopoiesis, resulting in higher numbers of CD11b^+^ myeloid cells in the BM.

### ILC2s induce HSPC proliferation and differentiation via soluble factors

**Figure 3:**
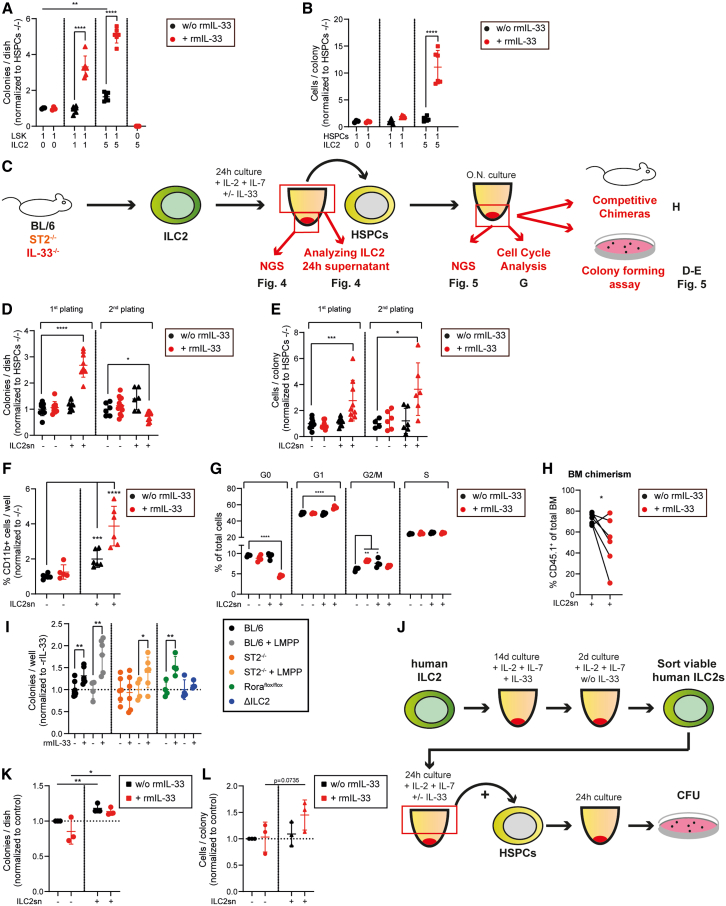
IL-33-activated ILC2s increase the colony-forming capacity of HSPCs and induce myeloid differentiation (A and B) Colony forming assay performed with 1 × 10^3^ FACS-purified BL/6 HSPCs (1). Before plating HSPCs in methylcellulose, they were co-cultured O.N. with 1 × 10^3^ (1) or 5 × 10^3^ (5) FACS-purified BL/6 ILC2s (*n* = 6) or without ILC2s (*n* = 3), either in the absence or presence of rmIL-33 (100 ng/ml). Colonies (A) and cells per colony (B) were counted 7 days later. 5 × 10^3^ ILC2s (*n* = 5) were plated without HSPCs as negative controls. Data are normalized to the control condition (no ILC2s, no rmIL-33). (C) Experimental layout of the ILC2 supernatant (ILC2sn) and HSPC interaction experiments. BL/6, ST2^−/−^, IL-33^−/−^ were FACS-purified from BM of naive mice and cultured for 24 h in media supplemented with rmIL-2 (50 U/ml) and rmIL-7 (50 ng/ml), with or without rmIL-33 (10 ng/ml). ILC2 supernatant (ILC2sn) was harvested and used for O.N. culturing of HSPCs. (D and E) Colony forming assay performed with 1 × 10^3^ FACS-purified HSPCs. Before plating in methylcellulose, HSPCs were cultured O.N. in Ctrl, Ctrl + IL-33, ILC2sn, or IL-33-stim. ILC2sn medium (*n* = 9 for the first plating; *n* = 6 for the second plating; pooled data from 2 independent experiments). Colonies and cell numbers were counted 7 days later, and 1 × 10^4^ cells were replated. Replated colonies were counted 14 days post-seeding. Data are normalized to the control condition (no ILC2s, no rmIL-33). (F) 5 × 10^3^ FACS-purified HSPCs were cultured for 4 days in control medium or ILC2 supernatant before analysis of the frequency of CD11b^+^ cells by FACS (*n* = 6 per condition; data pooled from 2 independent experiments). (G) Analysis of the cell cycle phases of Ctrl, Ctrl + rmIL-33, ILC2sn, and IL-33-stim. ILC2sn cultured HSPCs (O.N.; *n* = 4 per condition). Cell cycle phases were measured by flow cytometry using the Ki67/DAPI staining and are represented as a percentage of total cells. (H) Chimera experiment; 5 × 10^3^ Ly5.1^+^ FACS-purified HSPCs were cultured in ILC2sn or IL-33-stim. ILC2sn O.N. (paired) and then transplanted with 2 × 10^5^ Ly5.2^+^ total BM cells into lethally irradiated CD45.2^+^ recipients (*n* = 6; data pooled from 2 independent experiments). Percentage of Ly5.1^+^ cells out of total BM cells was assessed 18 weeks post-transplantation. (I) Colony-forming assay performed with 1 × 10^3^ FACS-purified HSPCs from individual BL/6 (with or without LMPP transfer), ST2^−/−^ (with or without LMPP transfer), Rora^flox/flox^ or ΔILC2 mice, either treated or untreated with recombinant (r)IL-33 24 h before sacrifice. Colonies were counted 7 days post-plating in methylcellulose (*n* = 4–6 per condition; biological replicates). (J) Experimental layout of the human ILC2/HSPC interaction experiment. ILC2s were FACS-sorted from healthy individuals and cultured for 16 days in an expansion medium with rhIL-2 and rhIL-7 in the presence of rhIL-33 for the first 14 days and then without rhIL-33 for the last 2 days. After that, viable ILC2s were FACS-sorted and plated to produce ILC2 supernatant (ILC2sn). (K and L) Colony forming assay performed with 1 × 10^3^ FACS-purified human HSPCs. Before plating in methylcellulose, HSPCs were cultured for 24 h in Ctrl, Ctrl + IL-33 medium, or ILC2sn (with or without rhIL-33). Each data point represents the average colony count of a biological replicate (*n* = 3). The dotted line represents the normalized colony count of the control medium cultured HSPCs. Colonies and cell numbers were counted 7–14 days post-plating. Data are represented as mean ± SD (A, B, D, E, F, G, I, K, L). Statistics: two-tailed Student’s t test (A, B, H, I, K, and L); one-way ANOVA (D, E, F, and G); ∗*p* < 0.05, ∗∗*p* < 0.01, ∗∗∗*p* < 0.001, ∗∗∗∗*p* < 0.001. See also Figure S3.

Next, we studied the mechanisms by which ILC2s regulate myelopoiesis by analyzing the effect of ILC2s on the colony-forming capacity of HSPCs *in vitro*. We co-incubated BL/6 ILC2s and HSPCs with or without rmIL-33 overnight (O.N.) before culturing them in methylcellulose (Figure 3A). Consistent with the observation that HSPCs did not express ST2 (Figure S1D), rmIL-33 did not alter the colony-forming capacity of HSPCs. Co-culturing of HSPCs with ILC2s (ratio 1:5) without rmIL-33 resulted in a minor increase in colony numbers. However, co-culturing HSPCs with rmIL-33 stimulated ILC2s significantly increased colony formation and resulted in higher cell numbers per colony (Figure 3B). Previous studies have shown that ILC2s produce large amounts of cytokines to communicate in a paracrine fashion with other cells.^50^ To test whether ILC2s regulate HSPCs via soluble factors, we incubated HSPCs with IL33-stimulated or unstimulated ILC2 supernatants (IL-33-stim. ILC2sn vs. ILC2sn) before culturing them in methylcellulose (Figure 3C). Pre-incubation of HSPCs with IL-33-stim. ILC2sn increased the colony number and the number of cells per colony compared to HSPCs pre-incubated with ILC2sn (Figures 3D and 3E). In the second plating, HSPCs pre-cultured with IL-33-stim. ILC2sn formed fewer, but larger colonies compared to the control conditions. In addition, pre-incubation of HSPCs with ILC2sn from both, IL-33-stim. and non-stimulated ILC2s, increased the percentage of CD11b^+^ cells compared to the control conditions without ILC2sn (Figure 3F). This indicates that pre-incubation with IL-33-stim. ILC2sn expands HSPCs with increased myeloid differentiation capacity.

Next, we analyzed the cell cycle phases of the HSPCs that were cultured with or without ILC2sn. IL-33-stim. ILC2sn induced a shift from the G0 to the G1 phase compared to the control conditions (Figure 3G). rmIL-33 treatment alone slightly increased the percentage of cells in the G2/M phase. This observation may be explained by the fact that some HSPCs differentiated into CMPs, which expressed ST2 at low levels and, therefore, were sensitive to IL-33. To test the *in vivo* BM reconstitution potential of Ly5.1^+^ HSPCs that were pre-conditioned with ILC2sn or IL-33-stim. ILC2sn *in vitro*, we transplanted HSPCs together with Ly5.2^+^ rescue BM cells into lethally irradiated recipients. HSPCs pre-incubated with IL-33-stim. ILC2sn had a reduced capacity for long-term reconstitution compared to HSPCs pre-incubated with non-stimulated ILC2sn (Figure 3H). In summary, these results indicate that IL-33 stimulation of ILC2s induces proliferation and differentiation of HSPCs via release of soluble factors, resulting in reduced long-term reconstitution capacity of the HSPC compartment.

Next, we tested the effect of IL-33 on BM-resident ILC2s and HSPCs *in vivo*. First, we verified if the intraperitoneal injection of IL-33 increases the IL-33 BM concentration in IL-33^−/−^ mice (Figures S3A and S3B). To assess whether IL-33 regulates HSPC proliferation and differentiation via ST2-signaling in ILC2s, we injected rmIL-33 into LMPP-reconstituted BL/6 and ST2^−/−^ mice (Figure 2A). The injection of rmIL-33 increased the colony-forming capacity of HSPCs from LMPP-reconstituted BL/6 and ST2^−/−^ mice, whereas the colony-forming capacity of HSPCs derived from non-LMPP-reconstituted mice was only affected by the addition of rmIL-33 in BL/6 but not ST2^−/−^ mice (Figure 3I). Furthermore, rmIL-33 injection increased the colony-forming capacity of Rora^flox/flox^ HSPCs, while ΔILC2 HSPCs were unaffected (Figure 3I). These findings provide strong evidence that IL-33/ST2 signaling in ILC2s induces the expansion of HSPCs *in vitro* and *in vivo*.

Repetitive IL-33 injection increased the frequency, size, granularity, and expression of activation markers on ILC2s (Figures S3C–S3E).^51^ Furthermore, SSC-high CD11b^+^ myeloid cells accumulated in the BM of BL/6 and Rora^flox/flox^ mice upon IL-33 treatment (Figures S3F and S3G). This effect depended on ILC2s since ΔILC2 mice showed only a mild increase in this SSC-high CD11b^+^ population. As described before, IL-33 treatment led to splenomegaly, which was absent in ΔILC2 mice (Figure S3H).^28^ These findings indicate that IL-33 boosts myelopoiesis by activating BM-resident ILC2s.

To translate these findings from the mouse model to the human BM, we measured the effect of *in vitro* expanded ILC2s from healthy donors on the colony-forming capacity of HSPCs (Figures 3J and S3I). IL-33 did not change the colony-forming capacity of HSPCs (Figures 3K and 3L). However, HSPCs formed more colonies when cultured O.N. in ILC2sn and the IL-33-stim. ILC2sn increased the number of cells per colony.

Thus, murine and human ILC2s increase HSPC proliferation and differentiation by soluble factors.

### IL-33/ST2 signaling in ILC2s stimulates cytokine and chemokine secretion

**Figure 4:**
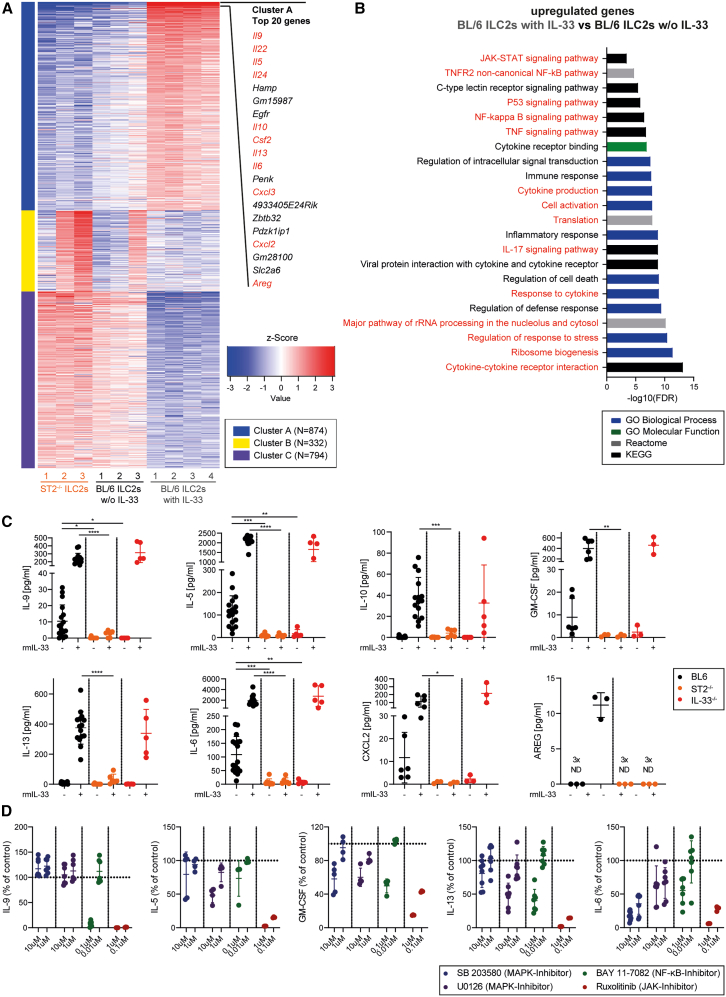
IL-33/ST2 signaling in ILC2 stimulates the secretion of various cytokines via the MAPK, NF-κB, and JAK-STAT pathways (A) K-means clustering of the top 2,000 differentially expressed genes (DEGs), received when comparing the RNAseq results from BL/6 ILC2s cultured for 24 h without or with rmIL-33 (BL/6 ILC2s w/o IL-33, and BL/6 ILC2s with IL-33, respectively) and ST2^−/−^ ILC2s cultured without IL-33 (ST2^−/−^ ILC2s w/o IL-33). Three clusters were generated (A–C), and the top 20 DEGs from cluster A are displayed. (B) Gene ontology (GO) analysis of upregulated genes (BL/6 ILC2s with IL-33 vs. BL/6 ILC2s w/o IL-33). A GO enrichment score of ≥ 3 indicates significant changes in gene expression. (C) IL-9, IL-5, IL-10, GM-CSF, IL-13, IL-6, CXCL2, and AREG cytokine levels measured in the 24 h supernatant of FACS-sorted BL/6, ST2^−/−^, or IL-33^−/−^ ILC2s, cultured in presence or absence of rmIL-33 (10 ng/ml; *n* = 3–13 per group; data pooled from 7 independent experiments). Cytokine levels were measured with a U-Plex multiplex assay from MSD or an ELISA (AREG). (D) Relative IL-9, IL-5, GM-CSF, IL-13, and IL-6 levels, measured in the 24 h supernatant of ILC2s that were treated with SB 203580, U0126, BAY 11–7082, or Ruxolitinib (*n* = 3–7 per condition, pooled from 3 independent experiments). Data are represented as the percentage of the expression in the control condition (DMSO-only treated ILC2s). Data are represented as mean ± SD. Statistics: one-way ANOVA (C); ∗*p* < 0.05, ∗∗*p* < 0.01, ∗∗∗*p* < 0.001, ∗∗∗∗*p* < 0.0001. See also Figure S4.

To study the transcriptomic changes of BM-resident ILC2s induced by IL-33 stimulation, we performed RNA sequencing (RNA-seq) of BL/6 ILC2s that were cultured for 24 h with or without rmIL-33 and ST2^−/−^ ILC2s as a control. Principal-component analysis (PCA) revealed that the three cell populations clustered separately (Figure S4A). We identified 3,450 differentially expressed genes (DEGs) between all groups, with the largest differences appearing when we compared the rmIL-33 treated BL/6 ILC2s with the other two groups (Figure S4B). The top 2,000 DEGs were divided into three clusters (cluster A–C) by K-means clustering, with DEGs in cluster A being upregulated and DEGs in cluster C being downregulated in BL/6 ILC2s with IL-33 (Figure 4A). DEGs in cluster B were partially upregulated in ST2^−/−^ ILC2s. Eleven out of the top 20 DEGs in cluster A were cytokines or chemokines (*Il9*, *Il22*, *Il5*, *Il24*, *Il10*, *Csf2* (translated into GM-CSF), *Il13*, *Il6*, *Cxcl3*, *Cxcl2*, and *Areg*). Interestingly, BL/6 ILC2s w/o IL-33 expressed higher *Il22*, *Il5*, *Csf2*, *Il13*, *Cxcl3*, and *Areg* levels compared to ST2^−/−^ ILC2s, indicating that already without further *ex vivo* IL-33 stimulation, basal *in vivo* IL-33/ST2 signaling activated BM-resident ILC2s (Figure S4C).

Next, we performed a gene ontology (GO) analysis comparing BL/6 ILC2s stimulated with or without IL-33. The presence of rmIL-33-induced genes associated with ribosome biogenesis, rRNA processing, and translation in ILC2s indicates an increased protein synthesis in these cells (Figure 4B). Furthermore, ILC2s stimulated with IL-33 upregulated genes involved in stress regulation, cell activation, and cytokine production. Moreover, genes associated with NF-κB and JAK-STAT signaling pathways were upregulated, suggesting that these pathways are downstream mediators of IL-33/ST2 signaling in ILC2s.

To evaluate the physiological differences between naive BL/6 and ST2^−/−^ ILC2s without additional *in vitro* rmIL-33 stimulation, we performed a GO analysis comparing these two populations. Already in naive BL/6 ILC2s, genes involved in the positive regulation of cytokine production, in cell cycle, M Phase, G2/M checkpoints, and JAK-STAT signaling were increased in BL/6 compared to ST2^−/−^ ILC2s (Figure S4D). This suggests that basal IL-33 concentrations in the BM are sufficient to activate ILC2s in steady-state.

To confirm the data obtained by RNA-seq, we harvested the supernatant from BL/6, ST2^−/−^ and IL-33^−/−^ ILC2s treated with or without rmIL-33 and assessed their cytokines and chemokine secretory potential. Non-stimulated BL/6 ILC2s secreted increased levels of IL-9, IL-5, and IL-6 compared to non-stimulated ST2^−/−^ and IL-33^−/−^ ILC2s, indicating that in the naive BM, basal IL-33/ST2 signaling induces cytokine and chemokine secretion by ILC2s (Figure 4C). Addition of rmIL-33 induced a strong cytokine and chemokine release of BL/6 and IL-33^−/−^ ILC2s, while ST2^−/−^ ILC2s were not affected. AREG was only measurable in the supernatant of IL-33-stimulated BL/6 ILC2s but not in the supernatant of unstimulated ILC2s. Injecting IL-33 *in vivo* into BL/6 mice also induces ILC2s to secrete higher levels of IL-9, IL-5, IL-10, IL-13, and IL-6 (Figure S4E). Furthermore, IL-33 injection increases the levels of IL-9, IL-5, IL-10, and IL-6 in the BM fluid of IL-33^−/−^ mice (Figure S4F). In summary, we show that BM-resident ILC2s release a variety of chemokines and cytokines in response to IL-33/ST2 signaling.

### IL-33/ST2 signaling activates NF-ΚB, MAPK, and JAK-STAT pathways in ILC2s

To determine the underlying mechanisms by which IL-33/ST2 activates cytokine and chemokine production by BM-resident ILC2s, we performed a gene set enrichment analysis (GSEA). IL-33 treatment induced genes involved in MAPK4/6, canonical NF-κB, and JAK-STAT signaling in BL/6 ILC2s (Figure S4G). To evaluate whether these pathways indeed regulated cytokine and chemokine production in ILC2s, we treated fluorescence-activated cell-sorting (FACS)-sorted ILC2s with MAPK inhibitors (SB 203580 or U0126), an NF-κB inhibitor (BAY 11–7082) or a JAK-STAT inhibitor (Ruxolitinib), before adding rmIL-33 to the cell culture (Figure 4D). Interestingly, different pathways were essential for the release of specific cytokines. For example, IL-9 secretion was regulated by the NF-ΚB and JAK-STAT pathways, while IL-6 was under the control of all tested pathways. However, the JAK-STAT pathway was the most potent regulator of the expression of the analyzed cytokines. Taken together, these results indicate that IL-33/ST2 signaling activates the NF-κB, MAPK, and JAK-STAT pathways in ILC2s, leading to the production and release of effector cytokines.

### IL-6, GM-CSF, and AREG secreted by ILC2s induce proliferation and differentiation of HSPCs

**Figure 5:**
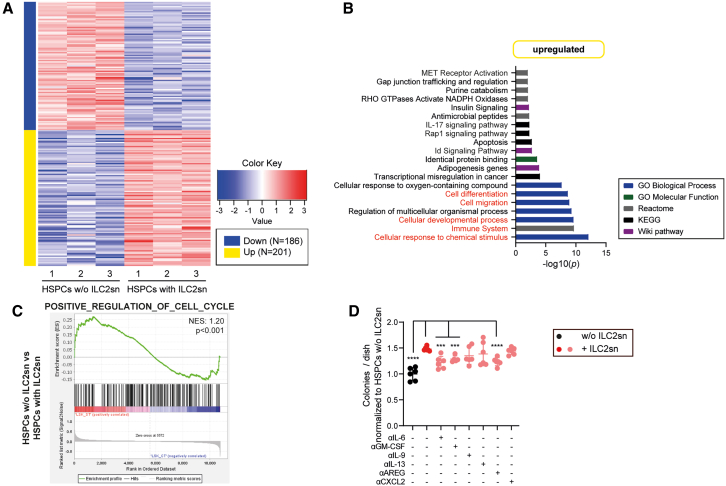
IL-6, GM-CSF, and AREG secreted by ILC2s induce proliferation and differentiation of HSPCs (A) Heatplot, representing the DEGs comparing HSPCs cultured with or without ILC2 supernatant (HSPCs w/o ILC2sn, HSPCs with ILC2sn). (B) Gene ontology (GO) analysis of upregulated genes (HSPCs with ILC2sn vs. HSPCs w/o ILC2sn). A GO enrichment score of ≥ 3 indicates significant changes in gene expression. (C) GSEA representing the enrichment score of the POSITVE_REGULATION_OF_CELL_CYCLE gene set, comparing HSPCs w/o ILC2sn with HSPCs with ILC2sn. (D) Colony forming assay performed with 1 × 10^3^ FACS-purified HSPCs. Before plating in methylcellulose, HSPCs were cultured O.N. with rmIL-33, or BL/6 ILC2sn, supplemented with different cytokine/chemokine neutralizing antibodies (αIL-6, αGM-CSF, αIL-9, αIL-13, αAREG, αCXCL2; *n* = 6 per condition; data are pooled from 2 independent experiments). Colonies were counted 7 days post-plating in methylcellulose. Data are represented as mean ± SD. Statistics: one-way ANOVA; ∗*p* < 0.05, ∗∗*p* < 0.01, ∗∗∗*p* < 0.001, ∗∗∗∗*p* < 0.0001. See also Figure S5.

To investigate how ILC2-secreted cytokines and chemokines affect HSPCs, we performed an RNA-seq analysis of HSPCs that were cultured for 24 h with IL-33-stimulated ILC2sn or control medium only. PCA analysis revealed that the two experimental HSPC groups formed distinct clusters (Figure S5A). In total, we identified 387 differentially expressed genes between HSPCs incubated with versus without ILC2sn; 201 genes were upregulated and 186 genes were downregulated in ILC2sn pre-conditioned HSPCs (Figure 5A). GO analysis revealed that the ILC2sn-induced genes were involved in the immune system and the cellular response to chemical stimuli, indicating that HSPCs undergo activation upon treatment with ILC2sn (Figure 5B). Furthermore, DEGs related to the cellular developmental, cell migration, and cell differentiation processes were upregulated in HSPCs treated with ILC2sn. GSEA revealed that genes involved in the positive regulation of the cell cycle were upregulated in HSPCs treated with ILC2sn (Figure 5C). These results support our finding, showing that ILC2sn induced differentiation and proliferation of HSPCs. No clear expression pattern was detectable for the downregulated DEGs when performing a GO analysis (Figure S5B).

To determine which of the secreted cytokines are responsible for the induction of differentiation and proliferation in HSPCs, we incubated HSPCs O.N. with a cocktail of selected cytokines that have been identified in ILC2sn and performed a colony formation assay (Figure S5C). We found that the colony formation capacity of HSPCs was increased when they were pre-conditioned with the cytokine cocktail. The omission of individual cytokines revealed that rmIL-6 was critical for the increase in colony numbers. Next, we pre-incubated HSPCs with ILC2sn in the presence or absence of cytokine/chemokine-neutralizing antibodies. We found that the colony-forming capacity was only reduced when αIL-6, αGM-CSF, and αAREG antibodies were added (Figure 5D). The number of cells per colony was reduced by αIL-6 and αGM-CSF treatment (Figure S5D).

These data demonstrate that IL-6, GM-CSF, and AREG secreted by ILC2s are key cytokines that drive the expansion of HSPCs while IL-6 and GM-CSF induce myeloid differentiation.

### IL-33/ST2 signaling induces the expansion of functionally impaired HSPCs after irradiation

**Figure 6:**
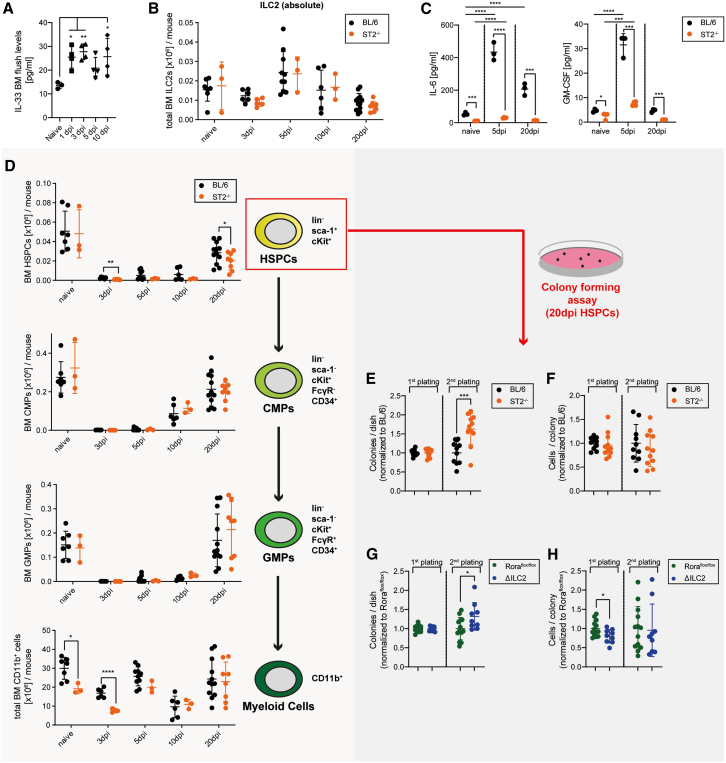
IL-33/ST2 signaling and ILC2s induce the expansion of HSPCs post-irradiation (A) IL-33 levels measured in the BM fluid of naive and sub-lethally irradiated BL/6; dpi = days post-irradiation (n = 3–4 per condition). (B) Total BM ILC2s in naive and sub-lethally irradiated BL/6 and ST2^−/−^ mice (*n* = 3–10 per condition; pooled from 2 independent experiments). (C) IL-6 and GM-CSF levels measured in the 24 h supernatant from BL/6 and ST2^−/−^ ILC2s, FACS-sorted from naive or sub-lethally irradiated mice (*n* = 3 per group). (D) HSPC, CMP, GMP, and myeloid cell numbers in naive and sub-lethally irradiated BL/6 or ST2^−/−^ mice (n = 3–10 per condition; pooled from 2 independent experiments). (E and F) Colony forming assay performed with 1 × 10^3^ HSPCs, FACS-sorted from BL/6 and ST2^−/−^ mice 20dpi plated in methylcellulose; 1 × 10^4^ cells were replated (*n* = 10–11; pooled from 2 independent experiments). (G and H) Colony forming assay performed with 1 × 10^3^ HSPCs, FACS-sorted from Rora^flox/flox^ control and ΔILC2 mice 20dpi plated in methylcellulose; 1 × 10^4^ cells were replated. Each dot represents one biological replicate (*n* = 9–13; pooled from 2 independent experiments). The colony counts in (E) and (G) are shown relative to the first plating. Data are represented as mean ± SD. Statistics: two-tailed Student’s t test (D, E, G, and H); one-way ANOVA (A and C); ∗*p* < 0.05, ∗∗*p* < 0.01, ∗∗∗*p* < 0.001, ∗∗∗∗*p* < 0.0001. See also Figure S6.

Irradiation is known to increase local tissue-specific IL-33 levels in skin, thymus, and spleen.^52^^,^^53^ In line with these findings, sublethal irradiation increased IL-33 levels in the BM day 1–10 post irradiation (dpi) (Figure 6A). Therefore, we next asked whether these elevated IL-33 levels could activate BM-resident ILC2s and regulate stress-induced hematopoiesis. Absolute numbers of ILC2 in BL/6 and ST2^−/−^ mice were stable pre- versus post-irradiation, indicating that ILC2s are radioresistant to the applied dose of 450 cGy and maintained independent of IL-33/ST2 signals (Figure 6B). Due to the increase in radiation-induced cell death of HSPCs and lineage^+^ hematopoietic cells, the frequency of ILC2s per total BM cells increased between days 3 and 10 post-irradiation (Figures S6A and S6B). Furthermore, irradiation induced IL-6 and GM-CSF secretion by ILC2s from BL/6 but not ST2^−/−^ mice (Figure 6C). In contrast, AREG was not secreted at detectable levels.

As expected, sub-lethal irradiation reduced the number of HSPCs, CMPs, GMPs, and CD11b^+^ myeloid cells (Figure 6D). Interestingly, HSPC numbers were significantly higher 20 days after irradiation in BL/6 compared to ST2^−/−^ mice, indicating that IL-33/ST2 signaling drives HSPC expansion post-irradiation. This was due to increased numbers of MPP2 and MPP3 in BL/6 mice compared to ST2^−/−^ animals 20 dpi, while the other stem and early progenitor cell types had comparable counts (Figure S6C). Functionally, FACS-purified BL/6 HSPCs 20 dpi formed slightly larger colonies in a first plating than ST2^−/−^ HSPCs (Figures 6E and 6F). In the second plating, the colony formation capacity of BL/6 HSPCs was reduced compared to ST2^−/−^ HSPCs. To analyze whether the HSPCs lose the capacity to self-renew after irradiation due to impaired ILC2 functionality, we repeated the colony formation experiment with ΔILC2 mice. HSPCs from 20 dpi ΔILC2 mice formed significantly smaller colonies compared to Rora^flox/flox^ control HSPCs in the first plating (Figures 6G and 6H). However, HSPCs from ΔILC2 mice had a higher re-plating capacity with a significant increase in colony numbers in the secondary plating. These results indicate that increased IL33 levels post-irradiation activate ILC2s that drive the expansion of HSPCs, resulting in a reduced self-renewal capacity.

### IL-33/ST2 signaling drives the expansion of functionally impaired and myeloid-skewed HSPCs during aging

**Figure 7:**
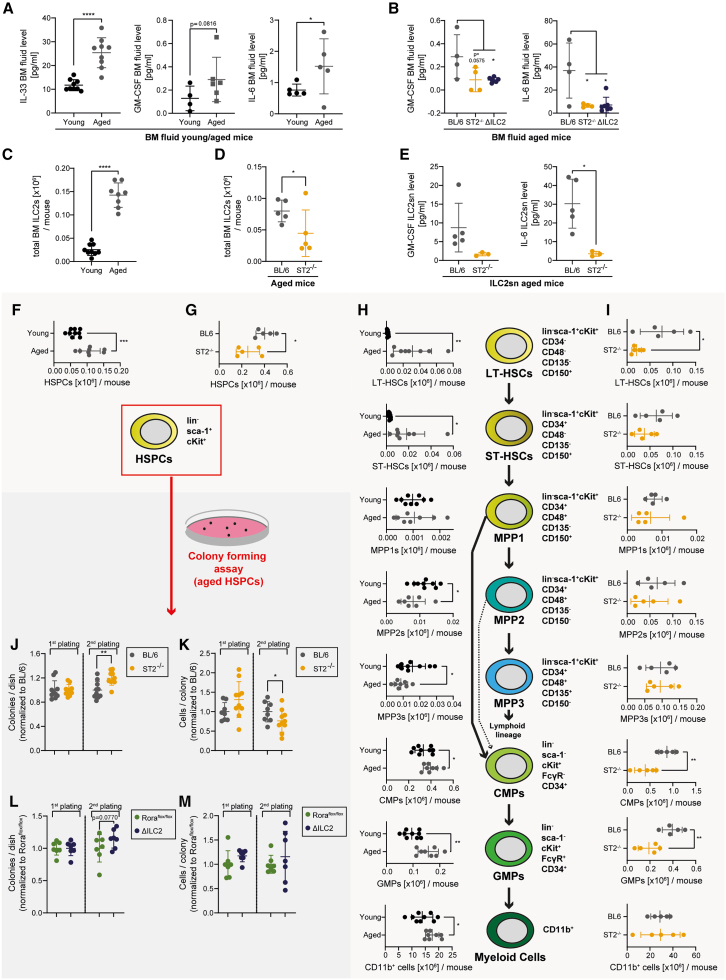
IL-33/ST2 signaling drives the expansion of functionally impaired and myeloid-skewed HSPCs during aging (A) IL-33, GM-CSF, and IL-6 BM fluid levels in young (2 months; *n* = 4–9) and aged (24 months; *n* = 5–8) BL/6 mice (data pooled from 3 independent experiments). (B) GM-CSF and IL-6 levels, measured in the BM fluid of aged BL/6 (*n* = 4), aged ST2^−/−^ mice (*n* = 4), and aged ΔILC2 (*n* = 7) mice. Each dot represents a physiological replicate. (C) Total BM ILC2 counts in young (*n* = 9) and aged BL/6 mice (*n* = 8; pooled from 2 independent experiments). (D) Total BM ILC2 counts in aged BL/6 (*n* = 5) and ST2^−/−^ mice (*n* = 5). (E) GM-CSF and IL-6 levels measured in the 24 h supernatant from aged BL/6 and ST2^−/−^ ILC2s. (F and G) HSPC counts in young (2 months; *n* = 9) vs. aged (24 months; *n* = 8 per group) BL/6 mice (F) and aged BL/6 vs. ST2^−/−^ mice (*n* = 5 per group; G). (H) LT-HSC, ST-HSC, MPP1, MPP2, MPP3, CMP, GMP, and CD11b^+^ myeloid cell BM cell numbers in young (*n* = 9) and aged (*n* = 8) BL/6 mice. (I) LT-HSC, ST-HSC, MPP1, MPP2, MPP3, CMP, GMP, and CD11b^+^ myeloid cell BM cell numbers in aged BL/6 (*n* = 5) and ST2^−/−^ (*n* = 5) mice. (J and K) Colony forming assay performed with 2 × 10^3^ HSPCs, FACS-sorted from aged BL/6 and ST2^−/−^ mice. 2 × 10^4^ cells were replated. Each dot represents one biological replicate (BL/6 *n* = 8; ST2^−/−^*n* = 10). (L and M) Colony forming assay performed with 2 × 10^3^ HSPCs, FACS-sorted from aged Rora^flox/flox^ control and ΔILC2 mice, plated in methylcellulose; 2 × 10^4^ cells were replated. Each dot represents one biological replicate (*n* = 7 per group). The colony counts in (J) and (L) are shown relative to the first plating. Data are represented as mean ± SD. Statistics: two-tailed Student’s t test; ∗*p* < 0.05, ∗∗*p* < 0.01, ∗∗∗*p* < 0.001, ∗∗∗∗*p* < 0.0001. See also Figure S7.

Since we observed that IL-33/ST2 signaling in BM-resident ILC2 promotes proliferation and myeloid differentiation in HSPCs under steady-state conditions, we sought to analyze the relevance of this process as a function of aging. Interestingly, IL-33, GM-CSF, and IL-6 levels in the BM of aged mice (22–24 months of age) were increased compared to young adult mice (8 weeks of age) (Figure 7A). ST2- and ILC2-deficiency in aged mice normalized the GM-CSF and IL-6 levels in the BM to levels of young adult mice (Figure 7B). AREG was not detectable in the BM fluid in all conditions. Moreover, ILC2 numbers were increased 15-fold in aged BL/6 mice compared to young mice (Figure 7C). In contrast, aged ST2^−/−^ mice had significantly reduced ILC2 counts in the BM compared to BL/6 mice, indicating that IL-33/ST2 signaling might be crucial for the maintenance or expansion of ILC2s during aging (Figure 7D). *Ex vivo*, ILC2s of aged mice produced similar amounts of GM-CSF and IL-6 on a per-cell basis compared to ILC2s of young mice, indicating that BM-resident ILC2s retain their functionality during the aging process (Figure S7A). Compared to ILC2s from aged BL/6 mice, ILC2s from aged ST2^−/−^ mice secreted less GM-CSF and IL-6 (Figure 7E).

In line with previous reports, we found that aged mice had increased HSPC counts (Figure 7F). This increase was absent in aged ST2^−/−^ mice compared to age-matched BL/6 controls (Figure 7G). Aging induced the accumulation of LT- and ST-HSCs, while MPP2s and MPP3s were less abundant compared to young controls (Figure 7H). Furthermore, aged mice had increased CMPs, GMPs, and CD11b^+^ myeloid cell counts. This phenomenon is also known as age-related myeloid skewing of hematopoiesis and is thought to contribute to decreased adaptive immune responses with age.^54^ Aged ST2^−/−^ mice had lower numbers of LT-HSCs and myeloid progenitors than aged BL/6 mice, suggesting that the age-related myeloid skewing is induced by IL-33/ST2 signaling (Figure 7I). Interestingly, colony-forming assays revealed a higher *in vitro* replating capacity of FACS-purified aged ST2^−/−^ HSPCs compared to BL/6 HSPCs. (Figures 7J and 7K). A similar increase in *in vitro* replating capacity was detectable in aged ΔILC2 HSPCs compared to Rora^flox/flox^ HSPCs (Figures 7L and 7M).

These data indicate that IL-33/ST2 signaling in ILC2s drives the aging process of the hematopoietic system with the typical expansion of functionally impaired HSCs and myeloid skewing.

## Discussion

BM resident immune cells crucially contribute to the regulation of hematopoiesis during homeostasis, but also after immune activation.^55^^,^^56^ Especially CD4^+^ T cells play an important role in maintaining HSC function after transplantation, mainly by producing hematopoiesis-promoting cytokines, such as IL-3 and GM-CSF.^12^ ILC2s are functionally comparable to T helper type 2 cells and produce large amounts of different cytokines. In contrast to CD4^+^ helper T cells that are activated by T cell receptor stimulation, ILCs respond to cytokines, such as IL-25 or IL-33. BM-resident ILC2s have been considered progenitor cells that lack the expression of the maturation marker KLRG1 and produce significantly fewer effector cytokines than ILC2s originating from barrier sites.^13^^,^^57^ However, recent studies showed that ILC2s differentiate early in the fetal liver or late in the periphery to become long-living tissue-resident cells.^13^^,^^58^^,^^59^ Therefore, the role of BM-resident ILC2s as progenitors for barrier sites in mature mice is under debate. The function of BM-resident ILC2s in hematopoiesis remains largely unknown. We now document that BM-resident ILC2s, in response to IL-33/ST2 signaling, crucially regulate the proliferation of HSPCs and myeloid differentiation during homeostasis, leading to functional alterations of aged HSCs with reduced self-renewal capacity and myeloid skewing. It has already been shown that ILC2s are potent regulators of various stem cell niches, for example, supporting HSC recovery and function in the BM or club cell regeneration after tissue injury in the lung.^15^^,^^25^^,^^60^^,^^61^^,^^62^

IL-33 is an alarmin that is released by various cell types upon stress or necrosis.^63^^,^^64^ However, our results indicate that IL-33 is secreted from BM-resident PαS stromal cells under homeostatic conditions. IL-33 levels in the BM were substantially higher than in blood, and IL-33 concentrations increased up to 3-fold during aging. Thus, in accordance with earlier studies, the BM is an IL-33-rich environment.^65^^,^^66^ Soluble IL-33 signals via its receptor ST2 that is expressed on different immune cells. Our detailed analysis of ST2 expression of BM-resident cells indicated that ILC2s highly express ST2 but that HSPCs are ST2 negative. The only hematopoietic cell population identified with detectable ST2 expression by flow cytometry was CMPs. In accordance with the absence of ST2, HSPCs did not respond to rmIL-33 *in vitro*, and BL/6 and ST2^−/−^ HSPCs engrafted similarly in competitive reconstitution experiments.

IL-33/ST2 signaling induced many molecular pathways involved in cell activation, protein production, and cytokine secretion. The release of these effector cytokines in ILC2s was induced by activating the JAK-STAT, NF-κB, and MAPK pathways. These pathways have already been demonstrated to be downstream of IL-33/ST2 signaling.^67^ Interestingly, the JAK-STAT pathway was involved in the production of all cytokines analyzed, whereas NF-κB and MAPK pathways selectively stimulated the production of defined cytokines. BM-resident ILC2s secrete cytokines under specific conditions, such as IL-5 in an IL-33-driven airway inflammation model or GM-CSF after 5-FU-treatment.^25^^,^^68^ By comparing BL/6 and ST2^−/−^ ILC2s, we demonstrated that even under steady-state conditions, BM-resident ILC2s secrete detectable IL-9, IL-5, GM-CSF, IL-6, and CXCL2 levels.

Many of the cytokines produced by ILC2s have well-known effects on hematopoiesis. For example, IL-6 derived from stromal fibroblasts reduces erythropoiesis but favors the expansion of GMPs.^69^ In addition, we documented that during a viral infection, cytotoxic T-cell (CTL)-secreted IFNy stimulates IL-6 production by MSCs that expands myeloid progenitors.^70^ Similarly, the GM-CSF receptor is expressed on HSCs and progenitors, and GM-CSF signaling expands myeloid progenitors, leading to increased myelopoiesis.^71^^,^^72^ Furthermore, it has been described that AREG, IL-9, and CXCL2 also can modify the HSPC function.^73^^,^^74^^,^^75^ Most likely, distinct cytokines secreted by ILC2s regulate HSCs concertedly. However, cytokine neutralization experiments and supplementation of individual cytokines point to a dominant role of IL-6, GM-CSF, and AREG in the expansion of HSCs and myeloid skewing. Importantly, ILC2s directly isolated from the BM without further IL-33 stimulation *in vitro* secreted IL-6 and GM-CSF, but not AREG, at detectable levels, and aged ST2^−/−^ and ΔILC2 mice had significantly lower concentrations of IL-6 and/or GM-CSF in BM.

The cytokines secreted by ILC2s induced molecular pathways in HSPCs associated with cell cycling, cell migration, and differentiation. Functionally, this was confirmed by a shift from G0 to the G1 state of HSPCs after incubation with cell supernatant from IL-33 stimulated ILC2s and an increased differentiation to CD11b^+^ myeloid cells. Although BM-resident ILC2s were already activated and secreted cytokines during homeostasis, the addition of IL-33 substantially increased cytokine production. Similarly, irradiation increased the IL-33 concentration in the BM and ST2 signaling in ILC2 induced IL-6 and GM-CSF production with consecutive expansion of HSPCs. This finding is in line with a recent study by Sudo et al. that documented that GM-CSF secreted by ILC2 supports hematopoietic recovery after 5-FU treatment.^25^ However, our *in vitro*-replating experiments indicate that although the number of phenotypically characterized HSPCs increases in the presence of ILC2s, the expanded HSPCs lose their capacity for self-renewal.

Thus, continuous IL-33/ST2 signaling in ILC2s, already under homeostasis but even more so after stress conditions, contributes to a functional decline of HSCs over time. Phenotypically characterized HSCs increase in numbers, and they upregulate CD150 expression (LT-HSCs) during aging. However, these HSCs are functionally impaired, as documented in single-cell and limiting dilution transplantation experiments, indicating a reduced self-renewal capacity and a myeloid skewing.^54^^,^^76^ We now document that ST2^−/−^ and ΔILC2 mice do not have these characteristic functional impairments of aged HSCs. The frequency of phenotypically characterized HSCs and LT-HSCs in ST2^−/−^ mice is lower, and the hematopoiesis is not skewed to the myeloid lineage, as shown by a significantly lower number of myeloid progenitors. Importantly, aged HSCs from ST2^−/−^ and ΔILC2 mice had a higher replating capacity *in vitro* than HSCs from BL/6 mice, suggesting a better self-renewal capacity. The aging-associated myeloid dominance in the BM is associated with elevated levels of pro-inflammatory cytokines, such as IL-6, TNF-α and IL-1Rα, a process known as “inflamm-aging”.^76^ These pro-inflammatory cytokines are produced by myeloid cells and adipocytes that increase in the aged BM. We have now documented increased levels of IL-33 in the aged BM and, as described before, increased numbers of ILC2s.^77^ Importantly, the increase of ILC2s during aging was dependent on an intact IL-33/ST2 signaling axis. Thus, elevated IL-33 concentrations contributed to an increase in cytokine concentrations in the BM of aged mice by increasing the number of cytokine-secreting ILC2s. Even though the absence of IL-33/ST2 signaling or ILC2s in full knockout (KO) mice improved HSC function and reduced the myeloid-skewing in aged animals, this does not necessarily lead to an improved immune control against infections in these animals. IL-33/ST2 signaling in effector cells is an essential regulator of immune responses against helminth,^78^^,^^79^ fungal,^80^ bacterial,^81^^,^^82^ or viral infections,^15^^,^^83^ either by directly participating in the immune response against the infectious trigger or by driving repair mechanisms after clearance of the infection.

In summary, IL-33/ST2 signaling in ILC2s leads to the production of hematopoietic cytokines that activate HSCs and induce cell division and myeloid differentiation. This pathway leads to increased numbers of HSCs and supports myelopoiesis after genotoxic stress, such as irradiation or chemotherapy, when IL-33 acts as an alarmin. However, chronic IL-33/ST2 signaling during aging leads to functionally impaired HSCs with reduced self-renewal and myeloid skewing. Thus, the “alarmin” IL-33 contributes to “inflamm-aging” of HSCs by stimulating the cytokine secretion of BM-resident ILC2s.

### Limitations of the study

This study provides evidence that BM-resident ILC2s regulate HSCs by inducing proliferation and myeloid differentiation. The study’s main limitation is that most of the experiments have been performed using mouse models or murine cells. We provided little evidence that human ILC2s can modulate hematopoiesis and no information about the exact molecular mechanisms in human HSCs. Another key limitation is that we showed the loss of stemness of phenotypically characterized HSCs in aged animals *in vitro* but not *in vivo* by serial re-transplantation assays.

## Resource availability

### Lead contact

Requests for further information and resources should be directed to and will be fulfilled by the lead contact, Adrian F. Ochsenbein (adrian.ochsenbein@insel.ch).

### Materials availability

This study did not generate new unique reagents.

### Data and code availability

All data reported in this paper will be shared with the lead contact upon request. All original sequencing data have been made publicly available on the GEO repository (https://www.ncbi.nlm.nih.gov/geo/query/acc.cgi?acc=GSE251700; https://www.ncbi.nlm.nih.gov/geo/query/acc.cgi?acc=GSE250530). Any additional information needed to reanalyze the data reported in this paper will be shared with the lead contact upon request.

## Acknowledgments

We thank the staff of Flow cytometry facility (FACSlab, University Bern, Switzerland) for providing technical assistance. Furthermore, we would like to thank Prof. Dr. Daniel Pinschewer (Experimental Virology, University of Basel, Switzerland) for providing IL-33^−/−^ and ST2^−/−^ mouse strains that have been backcrossed to BL/6 background. This work was supported by grants from Swiss Cancer Research (KFS-3815-02-2016) and Swiss National Science Foundation (SNF-310030-192675) to A.F.O.

## Author contributions

Conceptualization, P.N., A.F.O., C.A.J.-R., and C.R.; methodology, P.N., A.F.O., C.A.J.-R., and C.R.; investigation, P.N., N.S., S.F., I.M., D.B., and A.E.; visualization, P.N.; funding acquisition, A.F.O.; project administration, P.N.; supervision, A.F.O.; resources, A.F.O. and K.K.; writing – original draft, P.N. and A.F.O.; writing – review and editing, P.N. and A.F.O.

## Declaration of interests

The authors declare no competing interests.

## STAR★Methods

### Key resources table

**Table:** 

REAGENT or RESOURCE	SOURCE	IDENTIFIER
**Antibodies**		

FITC anti-mouse CD11c (N418)	Biolegend	Cat#117305; RRID: AB_313774
FITC anti-mouse CD127 (A7R34)	Biolegend	Cat#135007; RRID: AB_1937231
FITC anti-mouse CD34 (RAM34)	eBioscience	Cat#11-0341-82; RRID: AB_465021
PE anti-mouse CD25 (PC61)	eBioscience	Cat#12-0251-82; RRID: AB_465607
PE anti-mouse CD11b (M1/70)	eBioscience	Cat#12-0112-82; RRID: AB_2734869
PE anti-mouse CD45.1 (A20)	eBioscience	Cat#12-0453-82; RRID: AB_465675
PE anti-mouse CD45.2 (104)	Biolegend	Cat#109807; RRID: AB_313444
PE anti-mouse CD135 (A2F10)	Biolegend	Cat#135305; RRID: AB_1877217
PE anti-mouse cKit/CD117 (2B8)	Biolegend	Cat#105807; RRID: AB_313216
PE anti-mouse CD140α (APA5)	eBioscience	Cat#A18351; RRID: AB_2535212
PE anti-mouse CD48 (HM48-1)	Biolegend	Cat#103405; RRID: AB_313020
PE Ki-67 (SolA15)	eBioscience	Cat#12-5698-82; RRID: AB_11150954
PerCP-Cy5.5 anti-mouse sca-1 (D7)	Biolegend	Cat#108123; RRID: AB_313020
PerCP-Cy5.5 anti-mouse CD45.1 (A20)	Biolegend	Cat#110727; RRID: AB_893346
PerCP-eFluor710 anti-mouse ST2 (RMST2-2)	eBioscience	Cat#46-9335-82; RRID: AB_2573883
PerCP-eFluor710 Rat IgG2a,κ Ctrl (eBR2a)	eBioscience	Cat#46-4321-82; RRID: AB_1834455
PE-Cy7 anti-mouse cKit/CD117 (2B8)	Biolegend	Cat#105813; RRID: AB_313222
PE-Cy7 anti-mouse CD11c (N418)	Biolegend	Cat#117317; RRID: AB_493568
PE-Cy7 anti-mouse CD45.1 (A20)	Biolegend	Cat#110729; RRID: AB_1134170
PE-Cy7 anti-mouse CD11b (M1/70)	Biolegend	Cat#101215; RRID: AB_312798
PE-Cy7 anti-mouse CD16/32/FcγR (93)	Biolegend	Cat#101317; RRID: AB_2104156
PE-Cy7 anti-mouse CD48 (HM48-1)	Biolegend	Cat#103423; RRID: AB_2075049
PE-Cy7 anti-mouse CD31 (390)	Biolegend	Cat#102417; RRID: AB_830756
APC anti-mouse CD45.1 (A20)	Biolegend	Cat#110713; RRID: AB_313502
APC anti-mouse sca-1 (D7)	Biolegend	Cat#108111; RRID: AB_313348
APC anti-mouse CD25 (PC61)	Biolegend	Cat#102011; RRID: AB_312860
APC anti-mouse CD135 (A2F10)	Biolegend	Cat#135309; RRID: AB_1953264
APC anti-mouse CD48 (HM48-1)	Biolegend	Cat#103411; RRID: AB_571996
APC anti-mouse ST2 (DIH9)	Biolegend	Cat#145305; RRID: AB_2561917
APC anti-mouse Podoplanin (8.1.1)	Biolegend	Cat#127409; RRID: AB_10612940
eFluor450 anti-mouse CD34 (RAM34)	eBioscience	Cat#48-0341-82; RRID: AB_2043837
Pacific Blue anti-mouse CD150 (TC15-12F12.2)	Biolegend	Cat#115923; RRID: AB_2187962
Alexa Fluor 700 anti-mouse CD45.2 (104)	Biolegend	Cat#109821; RRID: AB_493730
Alexa Fluor 700 anti-mouse CD48 (HM48-1)	Biolegend	Cat#103425; RRID: AB_10612755
Alexa Fluor 700 anti-mouse CD90.2 (30-H12)	Biolegend	Cat#105319; RRID: AB_493724
APC-Cy7 anti-mouse cKit/CD117 (2B8)	Biolegend	Cat#105825; RRID: AB_1626278
APC-Cy7 anti-mouse Ep-CAM/CD326 (G8.8)	Biolegend	Cat#118217; RRID: AB_1501158
APC-Cy7 anti-mouse sca-1 (D7)	Biolegend	Cat#108125; RRID: AB_10645327
V500 Streptavidin	BD Biosciences	Cat#561419; RRID: AB_10611863
BV711 anti-mouse CD127 (SB/199)	BD Biosciences	Cat#565490; RRID: AB_2732059
Biotin anti-mouse CD19 (6D5)	Biolegend	Cat#115503; RRID: 115503
Biotin anti-mouse CD3e (145-2C11)	Biolegend	Cat#100303; RRID: AB_312669
Biotin anti-mouse Ly-6G/C/Gr-1 (RB6-8C5)	Biolegend	Cat#108403; RRID: AB_313368
Biotin anti-mouse TER119 (Ter-119)	Biolegend	Cat#116203; RRID: AB_313704
Pacific Blue anti-human CD45 (HI30)	Biolegend	Cat#304021; RRID: AB_2174123
PE anti-human CD127 (A019D5)	Biolegend	Cat#351303; RRID: AB_10720185
FITC anti-human ST2 (B4E6)	MD Biosciences	Cat#101002F; RRID: AB_947548
PE-Cy7 anti-human CD38 (HIT2)	Biolegend	Cat#303515; RRID: AB_1279235
APC-Cy7 anti-human CD90 (5E10)	Biolegend	Cat#328131; RRID: AB_2566341
APC anti-human CD34 (s20016e)	Biolegend	Cat#378605; RRID: AB_3068132
Biotin anti-human CD2 (RPA-2.10)	Biolegend	Cat#300203; RRID: AB_314027
Biotin anti-human CD14 (HCD14)	Biolegend	Cat#325623; RRID: AB_2074052
Biotin anti-human CD16 (3G8)	Biolegend	Cat#302003; RRID: AB_314204
Biotin anti-human CD19 (HIB19)	Biolegend	Cat#302203; RRID: AB_314233
Biotin anti-human CD235 (HIR2)	Biolegend	Cat#306617; RRID: AB_2565773
Biotin anti-human CD3 (OKT3a)	Biolegend	Cat#317319; RRID: AB_10916519
Purified mouse αIL-6 antibody (MP5-20F3)	Biolegend	Cat#504501; RRID: AB_315335
Purified mouse αGM-CSF antibody (MP1-22E9)	Biolegend	Cat#505401; RRID: AB_315377
Purified mouse αIL-9 antibody (MM9C1)	Biolegend	Cat#659505; RRID: AB_2876741
Mouse αIL-13 antibody (8H8)	InvivoGen	Cat#mil13-mab9-02; RRID: AB_2722583
Mouse αAREG blocking antibody	R&D Systems	Cat#AF989; RRID: AB_2060663
Mouse αCXCL2 blocking antibody (40605)	R&D Systems	Cat#MAB452; RRID: AB_2230058

**Biological samples**		

Healthy adult bone marrow (see Table S1)	University of Bern	N/A

**Chemicals, peptides, and recombinant proteins**		

Anti-biotin microbeads	Miltenyi	Cat#130-090-485
Recombinant mouse IL-2	Prospec	Cat#CYT-370
Recombinant mouse IL-7	Prospec	Cat#CYT-372
Recombinant mouse IL-33	Prospec	Cat#CYT-655
Recombinant mouse SCF	Prospec	Cat#CYT-275
Recombinant mouse Flt3L	Prospec	Cat#CYT-340
Recombinant mouse IL-3	Prospec	Cat#CYT-371
Recombinant mouse IL-6	Prospec	Cat#CYT-350
Recombinant mouse IL-9	Prospec	Cat#CYT-373
Recombinant mouse IL-13	Prospec	Cat#CYT-375
Recombinant mouse AREG	R&D Systems	Cat#989-AR-100/CF
Recombinant mouse IL-5	Prospec	Cat#CYT-689
Recombinant human IL-2	Prospec	Cat#CYT-209
Recombinant human IL-7	Prospec	Cat#CYT 254
Recombinant human IL-33	Prospec	Cat#CYT 425
Recombinant mouse IL-33 Protein (for *in vivo* experiments)	R&D Systems	Cat#3626-ML-010/CF
SB203580	alomone labs	Cat##S-370
U0126	alomone labs	Cat##U-400
BAY 11-7082	Sigma-Aldrich	Cat#B5556
Ruxolitinib	InvivoGen	Cat#tlrl-rux
Mouse Methocult Base Medium	Stemcell Technologies	Cat#M3134
StemSpan™ CC110	Stemcell Technologies	Cat#02697
StemSpan™-XF	Stemcell Technologies	Cat#100-0073
Human Methcult H4435 enriched	Stemcell Technologies	Cat#H4435

**Critical commercial assays**		

U-Plex assay (mouse IL-5, IL-6, IL-9, IL-13, IL-33, CXCL2, GM-CSF)	Meso Scale Discovery	N/A (individual quotes)
AREG Elisa Kit	R&D Systems	Cat#DY989

**Deposited data**		

ILC2 sequencing data	This paper	GEO: GSE251700
HSPC sequencing data	This paper	GEO: GSE250530

**Experimental models: Organisms/strains**		

Mouse: Il1rl1^tm1Anjm^ (ST2^-/-^)	Townsend et al.^38^	N/A
Mouse: Il33^tm1Snak^ (IL-33^-/-^)	Oboki et al.^84^	N/A
Mouse: C57BL/6J/Rora^fl/+^ x Il7r^tm1.1(icre)Hrr^ (Rora^+/flox^Il7r^Cre^)	Oliphant et al.^47^	N/A

**Software and algorithms**		

FlowJo v.10	BD Biosciences	https://www.flowjo.com/
iDEP.93	Ge et al.^85^	http://bioinformatics.sdstate.edu/idep93/
ShinyGO	Ge et al.^86^	http://bioinformatics.sdstate.edu/go77/
GSEA	Subramanian et al.^87^ Mootha et al.^88^	https://www.gsea-msigdb.org/gsea/index.jsp

### Experimental model and study participants details

#### Study design

This study aimed to understand the interactions between BM-resident ILC2s and HSPCs and how this interaction regulates hematopoiesis at steady-state, post-irradiation, or in aged subjects. We functionally addressed the role of ILC2s in hematopoiesis using different genetically modified mouse strains (ST2^-/-^, IL-33^-/-^, and Rora^flox/flox^ x IL7RCre mice). The animal experiments and protocols were performed according to the Swiss law for animal protection and approved by the local experimental animal committee of the Canton of Bern. All animal experiments were performed in 6–12-week-old or 18-24-month-old mice (if not further indicated females) housed in a specific pathogen-free facility in individually ventilated cages. Mice were randomly assigned to the experimental groups and, if possible, mixed before experiments to avoid cage effects. The Ethics Committee of the Canton of Bern approved the use of human specimens for this study. Human samples were obtained from the University Hospital Bern, Switzerland. All experiments were conducted and analyzed nonblinded; no animal or human sample was excluded. Experiments were performed multiple times in different independent replicates. *P* values were calculated using the method specified in the figure legends.

#### Mice

C57BL/6J (BL/6) and Ly5.1 mice were purchased from Charles River (Sulzfeld, Germany) or from Janvier Labs (Le Genest-Saint-Isle, France). ST2^-/-^ and Rora^flox/flox^ x IL7RCre mice were kindly provided by McKenzie A. (MRC Laboratory of Molecular Biology, Cambridge, UK).^38^^,^^47^ IL-33^-/-^ mice were kindly provided by Furuta Y. (RIKEN Center for Life Sciences Technologies, Kobe, Japan).^84^ Rag1^-/-^ x Ly5.1 mice were obtained from the Swiss Immunological Mouse Repository (SWIMR).^89^ ST2^-/-^, Rora^fllox/flox^ x IL7RCre, IL-33^-/-^, Ly5.1, and Rag1^-/-^ x Ly5.1 mice were all on a C57BL/6J background that was confirmed by SNP analysis performed at Taconic (Taconic Biosciences, Rensselaer, USA). Ly5.1 and ST2^-/-^ mice were crossed in-house to generate ST2^-/-^ x Ly5.1 animals. Female mice aged 6-12 weeks were used for experiments if not indicated otherwise in the Figure legend. Applications of animal experiments were performed according to Swiss laws for animal protection and approved by the local experimental animal committee of the Canton of Bern (BE75/17, BE78/17, BE13/2021, BE30/2021).

#### Patient samples

BM from healthy donors was collected from orthopedic patients who underwent vertebroplasty. The local ethical committee of the Canton of Bern, Switzerland (KEK 122/14 and BE2019-01627) approved the analysis of BM samples. The details about the patient samples are listed in Table S1. Based on the ethical approvals, we only received information on the age and gender of the orthopedic patients, but no information about race and ethnicity. For patient sample #1, no information about the age and gender of the patient has been transmitted from the clinics to our laboratory.

### Method details

#### Irradiation

Mice were sub-lethally (1 × 450cGy) or lethally (2 × 650cGy, with a 4h interval) irradiated with a Gammacell 40 extractor (MDS Nordion, Best Theratronics, Ottawa, Canada). After lethal irradiation, 200’000 total BM cells were injected into the mice (rescue BM), and the mice were treated with antibiotics for 2 weeks (Sulfamethoxazol and trimethoprim, Nopil, Mepha, 0.625mL/100mL of drinking water; Enrofloxacin (Baytril, Bayer, 0.15mL/100mL of drinking water).

#### Isolation and purification of primary murine hematopoietic cells

BM cells were harvested as described before.^90^ Long bones (femurs, tibiae, humeri) were collected from euthanized mice and flushed with MACS-buffer (consisting of phosphate-buffered saline (PBS), supplemented with 5% fetal calf serum (FCS) and 0.5% EDTA). The BM suspension was filtered through a 70μM cell strainer, and red blood cell lysis was performed. Total BM cell numbers were assessed with a Neubauer hemocytometer, and an aliquot for the BM lin^+^ staining was taken. Afterward, lineage-negative cells (BM lin^-^) were purified by magnetic cell separation (MACS) according to the manufacturer’s protocol. Therefore, BM cells were incubated with biotinylated antibodies against defined lin^+^ populations (αCD19, αCD3, αLy6G/C, αTER119). After washing, the biotin-labeled BM cells were incubated with anti-biotin microbeads (Miltenyi Biotec, Bergisch Gladbach, Germany), and finally, the lineage separation was performed using the autoMACS® Pro Separator (Miltenyi Biotec).

Long bones were flushed with 400μL (when femurs, tibiae, and humeri were used) or 250μL (when femurs and tibiae were used) ice-cold PBS to harvest the BM fluid. After centrifugation, the supernatant was harvested and stored at −20°C for analysis.

#### Isolation and purification of primary murine BM stroma cells

Isolation of BM stroma cells was performed as described before.^91^ Long bones were flushed, then crushed, cut into tiny pieces, and digested in a collagenase mix (0.166 mg/mL DNase, 1mg/ml Collagenase I, 2.5mg/ml Collagenase II in DMEM 4%; for 60min at 37°C on a shaker). After digestion, MACS buffer was added to the collagenase mix, and the cell suspension was filtered using a 40μM cell strainer, and collagenase activity was stopped by placing the samples on ice. Finally, red blood cell lysis was performed before the cells were stained for flow cytometry.

#### Flow cytometry and FACS-purification

Surface staining was performed by incubating cells for 30 min at 4°C in MACS-buffer, supplemented with the antibodies of interest in the specific, titrated concentration. Murine BM-resident HSPCs, HSPC subsets, progenitors, and myeloid cells were characterized as previously described; the gating strategies are shown in Figure S1C, S1E, and S1F.^92^^,^^93^^,^^94^^,^^95^^,^^96^ BM ILC2s were defined as lin^−^Sca-1^+^cKit^−^CD90.2^+^CD25^+^CD127^+^ cells (Figure S1A). Mesenchymal stromal cell populations were characterized as described before.^97^

Human BM-resident HSPCs were characterized as previously described^98^; the gating strategy is shown in Figure S3I. Human ILC2s were defined as lin^−^CD45^+^CD127^+^ST2^+^ cells (Figure S3I). We defined human cells as lineage-positive when they expressed CD3, CD14, CD16, CD19, CD56, or CD235a.

Ki67 staining was used to analyze the cell cycle. Therefore, cells were fixed and stained with the Foxp3/Transcription Factor Staining Buffer Set (eBioscience, San Diego, USA), according to the manufacturer’s protocol. Before performing the flow cytometry analysis, cells were additionally stained with DAPI.

FACS analysis was performed on an LSR Fortessa cell analyzer or LSR II Flow cytometer (BD Bioscience, San Jose, USA). Cell-sorting was done using a BD FACS ARIA III (BD Bioscience, San Jose, USA). Data was analyzed with FlowJo software v.10 (FlowJo, USA).

#### Generation of chimeric mice

Competitive BL/6-ST2^-/-^ chimeras were generated by FACS-sorting lin^-^Sca-1^+^cKit^+^ HSPCs from Ly5.1^+^5.2^+^ BL/6 and Ly5.2^+^ ST2^-/-^ mice. HSPCs were injected intravenously (i.v.) in a 1:1 ratio (5 × 1.5 × 10^4^ HSPCs each) into lethally irradiated Ly5.1^+^ BL/6 or Ly5.1^+^ ST2^-/-^ recipients with 2x10^5^ total Ly5.1^+^ BL/6 BM cells (rescue BM). 18 weeks post-transplantation, recipients were sacrificed and the chimerism in blood and BM was assessed by flow cytometry.

To generate BL/6-IL-33^-/-^ chimeras, we injected i.v. 5 × 10^6^ total BM cells from BL/6 or IL-33^-/-^ donors into lethally irradiated BL/6 or IL-33^-/-^ recipients (without rescue BM). Ten weeks after transplantation, recipients were sacrificed, and the BM fluid was harvested and frozen for analysis.

ILC2 supernatant-treated HSPC chimeras were generated by FACS-purifying Ly5.1^+^ BL/6 HSPCs before culturing them O.N. with ILC2 supernatant (ILC2sn; see section “culturing of BM-resident ILC2s”). Afterward, 5 × 10^3^ of the ILC2sn-treated HSPCs were injected with 2x10^5^ Ly5.2^+^ BL/6 total BM cells into lethally irradiated Ly5.2^+^ BL/6 recipients. Eighteen weeks post-transplantation, recipients were sacrificed, and the BM and blood chimerism were assessed by flow cytometry.

#### Generation of LMPP transfer mice

Lymphoid-primed multipotent progenitor cells (lin^-^sca-1^+^cKit^+^CD135^+^ LMPPs; Figure S2A) were FACS-purified from Rag1^-/-^ x CD45.1 donors and transplanted into sub-lethally irradiated BL/6, ST2^-/-^, Rora^flox/flox^, or ΔILC2 recipients (1 × 10^4^ LMPPs per recipient).^49^ As controls, BL/6, ST2^-/-^, Rora^flox/flox^, or ΔILC2 mice were irradiated without LMPP transfer. 18 weeks post-transplantation, recipients and controls were sacrificed, and the BM compartment was analyzed. Some of the LMPP transferred mice were injected intraperitoneally (i.p.) with *in vivo* rmIL-33 (12μg/kg; R&D Systems, Minneapolis, USA) 24h before takedown.

#### Culturing of BM-resident ILC2s

ILC2s were FACS-sorted from the BM of BL/6, ST2^-/-^, and IL-33^-/-^ mice and cultured in IMDM 10% (Iscove’s Modified Dulbecco’s Medium, supplemented with 10% FCS, 1% L-Glutamine, and 1% penicillin-streptomycin), supplemented with rmIL-2 (50U/ml) and rmIL-7 (50ng/ml), in presence or absence of rmIL-33 (10ng/ml). Cells were plated in 96 U-well plates, with a density of 5 × 10^3^ cells per 100μL medium. After 24h of culturing, the supernatant (ILC2sn) was harvested and stored at −20°C for further experiments. To block the activity of selected cytokines/chemokines, ILC2sn was treated for 1h at 37°C with an αIL-6 antibody (10μg/ml; Biolegend, San Diego, USA), αGM-CSF antibody (10μg/ml; Biolegend, San Diego, USA), αIL-9 antibody (10μg/ml; Biolegend, San Diego, USA), αIL-13 antibody (1μg/ml, InvivoGen, San Diego, USA), αAREG (1μg/ml, R&D Systems, Minneapolis, USA), or an αCXCL2 antibody (1μg/ml, R&D Systems, Minneapolis, USA).

To inhibit specific pathways, ILC2s were cultured in the presence of small molecule inhibitors: SB 203580 or U0126, blocking the MAPK pathway (both from alomone labs, Jerusalem, Israel); BAY 11-7082 to block the NF-ΚB pathway (Sigma-Aldrich, St. Louis, USA), or Ruxolitinib to block the JAK-STAT pathway (InvivoGen, San Diego, USA). All inhibitors were diluted in 100% DMSO, preparing a 10mM stock solution. The inhibitors were added to ILC2s in the specific concentrations mentioned in the manuscript. The concentrations were chosen in a way that the ILC2s did not have an impaired viability compared to the control ILC2s. As a control, ILC2s were treated with DMSO only. After culturing ILC2s in the presence of small molecule inhibitors for 1h at 37°C, rmIL-33 (10μg/ml) was added, and ILC2s were incubated for an additional 22h at 37°C before the supernatant was collected and frozen for further analysis.

FACS-sorted human ILC2s were cultured in IMDM 10%, supplemented with rhIL-2 (50U/ml), rhIL-7 (10ng/ml) and rhIL-33 (50ng/ml) for 14 days. Afterward, we washed the ILC2s and re-plated them in IMDM 10% containing rhIL-2 and rhIL-7 for 2 days to rest. Then, viable ILC2s were FACS-sorted and plated in IMDM 10%, supplemented with rhIL-2 and rhIL-7, +/- rhIL-33. Cells were plated in 96 U-well plates, with a density of 5 × 10^3^ cells per 100μL medium. After 24h of culturing, the supernatant (ILC2sn) was harvested and directly used or stored at −20°C for further experiments.

#### Measuring cytokine concentrations in the BM fluid and the supernatant of ILC2 cultures

IL-5, IL-6, IL-9, IL-13, IL-33, CXCL2, and GM-CSF cytokine concentrations were measured using the U-Plex assay from Meso Scale Discovery (MSD, Rockville, USA) according to the manufacturer’s protocol. AREG cytokine concentrations were measured using the ELISA-Kit from R&D Systems (Minneapolis, USA).

#### Colony-forming cell (CFC) assays

Mouse CFC assays were performed as described before.^86^^,^^90^ FACS-purified HSPCs (1 × 10^3^ from young mice, or 2x10^3^ from aged mice) were plated in MethoCult medium (STEMCELL Technologies, Cambridge, USA), supplemented with 15% FCS, 20% BIT (consisting of 50mg/ml BSA, 1.44U/ml rh-insulin, and 250ng/ml human holo-transferrin in IMDM), 100μM 2-mercaptoethanol, 100U/ml penicillin, 100μg/ml streptomycin, 2mM L-glutamine, 50ng/ml rmSCF, 50ng/ml rmFLTL-3, 10ng/ml rmIL-3, and 10ng/ml rhIL-6. Depending on the experimental setting, rmIL-33 was supplemented (10ng/ml or 100ng/ml). 7 days post seeding, colony numbers were enumerated with bright field microscopy (Zeiss Axio Observer, 10x magnification). Cultures were washed with balanced salt solution (BSS), cells were counted, and 1 × 10^4^ cells were replated in methylcellulose medium.

The HSPC/ILC2 CFC assay was performed by co-culturing 1 × 10^3^ HSPCs with 1 × 10^3^ or 5 × 10^3^ FACS-sorted ILC2s O.N. in IMDM 10% medium, supplemented with rmIL-2 (50U/ml), rmIL-7 (50 ng/ml), rmSCF (50ng/ml), and rmFLTL-3 (50ng/ml). rmIL-33 (100ng/ml) was added to the culture if indicated. Then, cells were transferred into MethoCult medium as described above, without adding rmIL-3 or rhIL-6.

To perform ILC2sn CFC assays, 1-2x10^3^ FACS-purified HSPCs were cultured O.N. in a total volume of 100μL. 30μL consisted of ILC2sn or control medium supplemented with rmIL-2 (50U/ml), rmIL-7 (50 ng/ml), and if specified rmIL-33 (10 ng/ml or 100 ng/ml), the remaining 70μL consisted of IMDM 10% medium. In addition, rmSCF (50ng/ml) and rmFLTL-3 (50ng/ml) were added to the cultures. After O.N. culture, HSPCs were transferred to MethoCult medium as described above. rmIL-3 or rhIL-6 was not added in first but in second platings.

To mimic the cytokine composition of ILC2sn, a CFC assay was performed by incubating 1 × 10^3^ HSPCs O.N. in IMDM 10%, supplemented with rmSCF (50ng/ml), rmFLTL-3 (50ng/ml), rmIL-5 (600pg/ml), rhIL-6 (300pg/ml), rmIL-9 (90pg/ml), rmIL-13 (120pg/ml), and rmAREG (600pg/ml). To test the effect of individual cytokines, rmIL-5, rmIL-6, rmIL-9, rmIL-13 and rmAREG were omitted individually. All O.N. cultured HSPCs were transferred to MethoCult medium lacking rmIL-3 or rhIL-6.

The colony counts were corrected for potential differences in the first plating for the CFC assays performed with the irradiated and the aged HSPCs. Therefore, the colony counts were first normalized based on the control groups (BL/6 or Rora^flox/flox^, respectively). In the next step, the fold-change average from the first plating was calculated (average control group divided by the average of the experimental group), and the normalized colony counts (first and second plating) from the experimental group were multiplied with this fold-change average.

For human CFU assays, 1 × 10^3^ FACS-sorted HSPCs were cultured for 24h in a mix of 30μL StemSpan medium (STEMCELL Technologies, Cambridge, USA) and 70μL ILC2sn or control medium. The generation of human ILC2sn is described in detail in the ‘Culturing of BM-resident ILC2s’ section. As control medium, we used IMDM 10% supplemented with rhIL-2 (50U/ml) and rhIL-7 (10ng/ml), with or without rhIL-33 (50ng/ml). After the O.N. culture, we transferred the HSPCs into Methocult medium to grow colonies. Colonies were counted after 7-14 days.

#### High-throughput transcriptome analysis using next generation RNA sequencing (RNA-Seq)

ILC2s were FACS-purified from naïve BL/6 and ST2^-/-^ mice and cultured as described above. In total, 3 ILC2 groups were generated: BL/6 ILC2s cultured without rmIL-33, BL/6 ILC2s cultured with rmIL-33, and ST2^-/-^ ILC2s cultured without rmIL-33. HSPCs were FACS-purified from naïve BL/6 mice and cultured for 24h with rmIL-33 (10ng/ml) or rmIL-33 ILC2sn. Afterwards, cells were washed, resuspended in RLT lysis buffer (Qiagen, Hilden, Germany), and sent for RNAseq on dry ice to Novogene Europe (Cambridge, UK). RNA isolation, library preparation, and next generation sequencing were performed at Novogene.

A total amount of 1μg RNA per sample was used as input material for RNA sample preparations. NEBNext® UltraTM RNALibrary Prep Kit for Illumina® (NEB, USA) was used to generate libraries, and index codes were added to specific sequences to each sample, according to manufacturer’s protocol. Poly-T oligo-attached magnetic beads were used to gain mRNA from total RNA, using fragmentation. First strand cDNA was generated, using random hexamer primer and M MuLV Reverse Transcriptase, before the second strand cDNA was synthesized, using DNA Polymerase I and RNase H. Blunt end conversion of remaining overhangs was performed by using exonuclease/polymerase. Afterwards, 3’ ends of DNA fragments were adenylated, and hybridization was made by ligating NEBNext Adaptor with hairpin loop structure. To select cDNA fragments of 150∼200bp in length, the AMPure XP system was used to purify the library fragments (Beckman Coulter, Beverly, USA). The size-selected, adaptor-ligated cDNA was incubated at 37°C for 15min followed by 5min at 95°C with 3 μL USER Enzyme (NEB, USA). Then, PCR was done with Phusion High-Fidelity DNA polymerase, Universal PCR primers, and Index (X) Primer. Afterwards, PCR products were purified (AMPure XP system) and the Agilent Bioanalyzer 2100 system was used to determine library quality. The index-coded samples were clustered using a cBot Cluster Generation System and the PE Cluster Kit cBot-HS (Illumina), according to the manufacturer’s protocol. Finally, an Illumina platform was used to sequence the library preparations, and paired-end reads were generated.

#### RNA-Seq quality control, mapping, quantification, and data analysis

First, raw data (raw reads in FASTQ format) were processed through fastp. Raw reads containing adapter and poly-N sequences and low-quality raw reads were excluded (clean reads). Simultaneously, Q20, Q30, and GC content of the clean reads were calculated. Only clean data of high quality was used for the downstream analyses.

Reference genome files were directly downloaded from the genome website browser (NCBI/UCSC/Ensembl). Using the Spliced Transcripts Alignment to a Reference (STAR) software, paired-end clean reads were aligned to the reference genome.

The RNA-seq data were analyzed using the iDEP.93 online platform.^85^ Briefly, the raw read counts were uploaded, and the lowly expressed genes were removed (we kept genes with a minimal count per million (CPM) of 1.5 or higher in at least 3 libraries). In a next step, the count data were transformed for clustering and principal component analysis (PCA), using the EdgeR (log2(CPM+c)) algorithm. The pseudo count c was set at 4. For the k-means analysis of the ILC2 RNAseq data, the 2000 most variable genes were included and separated into 3 clusters. In a next step, the differentially expressed genes (DEGs) were identified using the DESeq2 method (ILC2 RNAseq: FDR cutoff 0.5, minimum fold change 2.5; HSPC RNAseq: FDR cutoff 0.05, minimum fold change 1.5).

#### Gene ontology and gene set enrichment analysis

For gene ontology (GO) analysis, the ShinyGO online platform was used.^86^ Gene set enrichment analysis (GSEA) was performed using the GSEA software from the Broad Institute.^87^^,^^88^

### Quantification and statistical analysis

Statistical analysis for functional studies, including flow cytometry, *in vitro* and *in vivo* data, was performed using GraphPad Prism v.8 (GraphPad Software, USA). The Shapiro-Wilk test was used to determine whether the data met the normality assumption. Data was analyzed using a two-tailed Student’s t test or one-way-ANOVA followed by Dunnett’s or Tukey’s post-test. Details on the quantification, normalization, and statistical tests used in every experiment can be found in the corresponding figure legend. All *p*-values were considered significant when *p* < 0.05. Data are displayed as mean ± SD. ∗*p* < 0.05; ∗∗*p* < 0.01; ∗∗∗*p* < 0.001, ∗∗∗∗*p* < 0.0001.
