# Supplementary material for: IL-33/ST2 signaling in ILC2s drives exhaustion and myeloid skewing of HSCs in response to hematopoietic stress and aging
Source: iScience. 2025 Apr 8;28(5):112378. doi: 10.1016/j.isci.2025.112378 (PMC12084005; doi:10.1016/j.isci.2025.112378)
Supplement: Document S1. Figures S1–S7 and Table S1 [file mmc1.pdf]

iScience, Volume *28*

Supplemental information

IL-33/ST2 signaling in ILC2s drives exhaustion and myeloid skewing of HSCs in response to hematopoietic stress and aging

Pascal Naef, Carla A. Jaeger-Ruckstuhl, Noah Schnüriger, Stefan Forster, Inês Monteiro, Daniel Brigger, Alexander Eggel, Kai Kessenbrock, Carsten Riether, and Adrian F. Ochsenbein

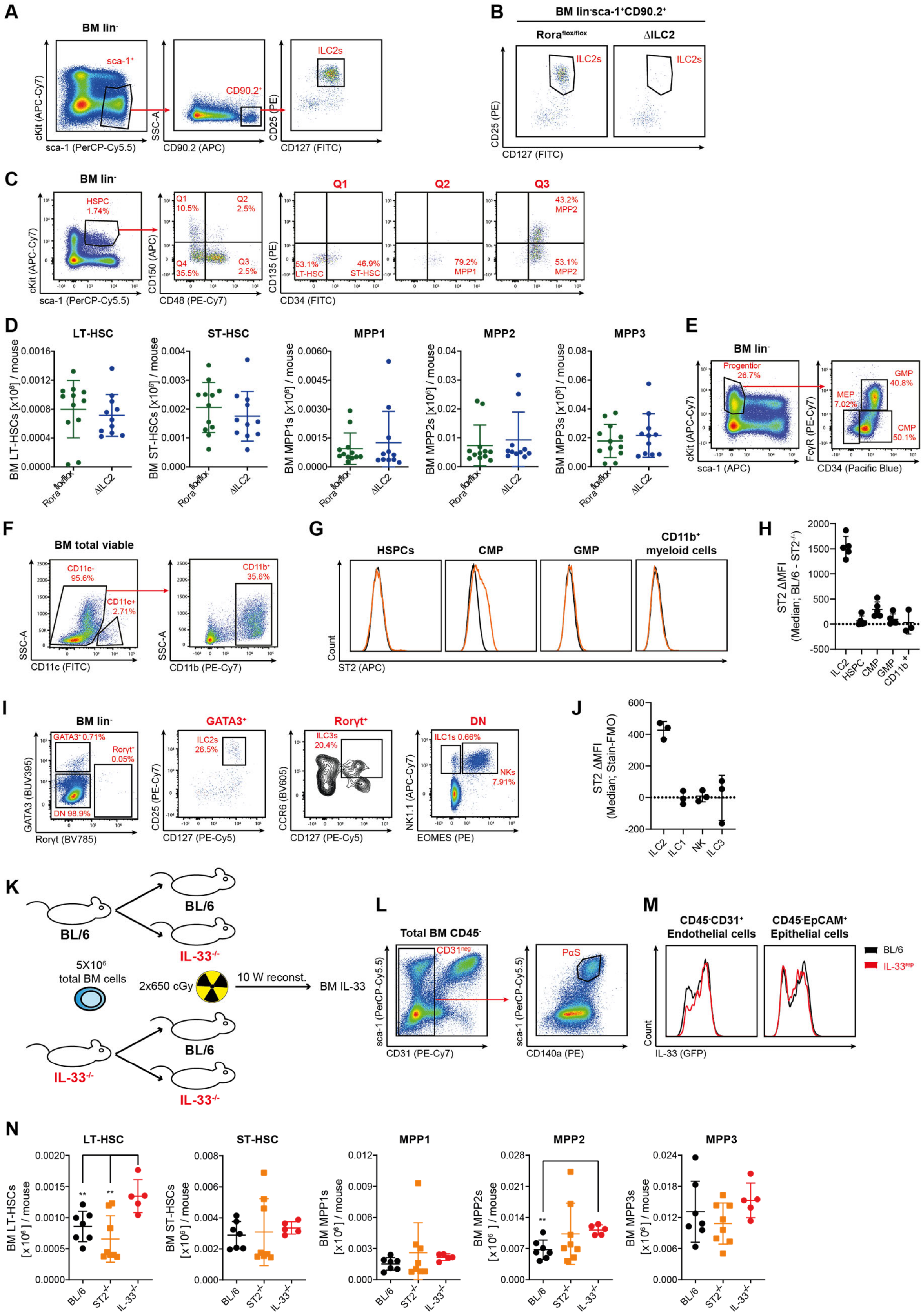

A
BM lin-
sca-1+
CD90.2+
ILC2s
cKit (APC-Cy7)
sca-1 (PerCP-Cy5.5)
SSC-A
CD90.2 (APC)
CD25 (PE)
CD127 (FITC)
B
BM lin-sca-1+CD90.2+
Rora flox/flox
ΔILC2
ILC2s
CD25 (PE)
CD127 (FITC)
C
BM lin-
HSPC 1.74%
Q1 10.5%
Q2 2.5%
Q3 2.5%
Q4 35.5%
Q1
Q2
Q3
53.1% LT-HSC
46.9% ST-HSC
79.2% MPP1
43.2% MPP2
53.1% MPP2
cKit (APC-Cy7)
sca-1 (PerCP-Cy5.5)
CD150 (APC)
CD48 (PE-Cy7)
CD135 (PE)
CD34 (FITC)
D
LT-HSC
ST-HSC
MPP1
MPP2
MPP3
BM LT-HSCs [x10^6] / mouse
BM ST-HSCs [x10^6] / mouse
BM MPP1s [x10^6] / mouse
BM MPP2s [x10^6] / mouse
BM MPP3s [x10^6] / mouse
Rora flox/flox
ΔILC2
E
BM lin-
Progenitor 26.7%
GMP 40.8%
MEP 7.02%
CMP 50.1%
cKit (APC-Cy7)
sca-1 (APC)
FcγR (PE-Cy7)
CD34 (Pacific Blue)
F
BM total viable
CD11c- 95.6%
CD11c+ 2.71%
CD11b+ 35.6%
SSC-A
CD11c (FITC)
CD11b (PE-Cy7)
G
HSPCs
CMP
GMP
CD11b+ myeloid cells
Count
ST2 (APC)
H
ST2 ΔMFI (Median; BL/6 - ST2-/-)
ILC2
HSPC
CMP
GMP
CD11b+
I
BM lin-
GATA3+ 0.71%
Rorγt+ 0.05%
DN 98.9%
GATA3+
ILC2s 26.5%
Rorγt+
ILC3s 20.4%
DN
ILC1s 0.66%
NKs 7.91%
GATA3 (BUV395)
Rorγt (BV785)
CD25 (PE-Cy7)
CD127 (PE-Cy5)
CCR6 (BV605)
NK1.1 (APC-Cy7)
EOMES (PE)
J
ST2 ΔMFI (Median; Stain-FMO)
ILC2
ILC1
NK
ILC3
K
BL/6
IL-33-/-
5X10^6 total BM cells
2x650 cGy
10 W reconst.
BM IL-33
L
Total BM CD45-
CD31neg
PαS
sca-1 (PerCP-Cy5.5)
CD31 (PE-Cy7)
CD140a (PE)
M
CD45-CD31+ Endothelial cells
CD45-EpCAM+ Epithelial cells
BL/6
IL-33rep
Count
IL-33 (GFP)
N
LT-HSC
ST-HSC
MPP1
MPP2
MPP3
BM LT-HSCs [x10^6] / mouse
BM ST-HSCs [x10^6] / mouse
BM MPP1s [x10^6] / mouse
BM MPP2s [x10^6] / mouse
BM MPP3s [x10^6] / mouse
BL/6
ST2-/-
IL-33-/-

Figure S1. IL-33/ST2 signaling in ILC2s promotes myelopoiesis, related to Figure 1.
(**A**) FACS-gating strategy for bone marrow (BM)-resident type 2 innate lymphoid cells (lin$^-$sca-1$^+$cKit$^-$ CD90.2$^+$CD25$^+$CD127$^+$ ILC2s). **(B)** FACS-gating for ILC2s in the BM of Rora$^{flox/flox}$ and Rora$^{flox/flox}$ x IL7RCre (ΔILC2) mice. **(C)** FACS-gating strategy for BM-resident hematopoietic stem and progenitor cells (HSPCs) and multipotent progenitor cells (MPPs). (**D**) Long-term hematopoietic stem cell (lin$^-$sca-1$^+$cKit$^+$CD150$^+$CD48$^-$CD34$^-$ LT-HSCs), short-term HSC (lin$^-$sca-1$^+$cKit$^+$CD150$^+$CD48$^-$CD34$^+$ ST-HSCs), and MPP (lin$^-$sca-1$^+$cKit$^+$CD150$^+$CD48$^+$CD34$^+$ MPP1s, lin$^-$sca-1$^+$cKit$^+$CD150$^-$CD48$^+$CD34$^+$CD135$^-$ MPP2s, and lin$^-$sca-1$^+$cKit$^+$CD150$^-$CD48$^+$CD34$^+$CD135$^+$ MPP3s) numbers in the BM of naïve Rora$^{flox/flox}$ (n=12) and ΔILC2 (n=11) mice. **(E)** FACS-gating strategy for BM-resident progenitor cells (common myeloid progenitors, CMPs; granulocyte-macrophage progenitors, GMPs; megakaryocyte-erythroid progenitors, MEPs). **(F)** FACS-gating strategy for BM-resident CD11b$^+$ myeloid cells. **(G)** ST2 expression on HSPCs, CMPs, GMPs, and myeloid cells, measured by flow cytometry; black: ST2$^{-/-}$ mouse; orange: BL/6. **(H)** Quantification (difference in mean fluorescence intensity, ΔMFI) of ST2-expression on ILC2s, HSPCs, CMPs, GMPs, and CD11b$^+$ myeloid cells, measured by flow cytometry (n=3-5, pooled from two independent experiments). ΔMFI was calculated by subtracting the MFI of ST2$^{-/-}$ cells from the MFI of BL/6 cells. **(I)** FACS-gating strategy for BM-resident ILC2s, type 3 innate lymphoid cells (ILC3s), type 1 innate lymphoid cells (ILC1s), and natural killer cells (NK cells). **(J)** Quantification (difference in mean fluorescence intensity, ΔMFI) of ST2-expression on ILC2s, ILC3s, ILC1s, and NK cells (n=3). **(K)** Experimental setup for the BL/6-IL-33$^{-/-}$ chimera experiment. 5x10^6 total BM cells from BL/6 or IL-33$^{-/-}$ donors were transplanted into lethally irradiated BL/6 or IL-33$^{-/-}$ recipients. **(L)** FACS-Gating strategy for BM-resident PDGFR-α$^+$sca-1$^+$ mesenchymal stromal cells (PαS cells). **(M)** IL-33 expression on BM-resident CD45$^-$CD31$^+$ endothelial cells and CD45$^-$EpCAM$^+$ epithelial cells, measured in BL/6 (black line) and IL-33-GFP-reporter mice (IL-33^{rep}; red line) by flow cytometry. **(N)** LT-HSC, ST-HSC, MPP1, MPP2 and MPP3 numbers in the BM of naïve BL/6 (n=7-10), ST2-knockout (ST2$^{-/-}$; n=8-11), and IL-33-knockout (IL-33$^{-/-}$; n=5) mice. Statistics: one-way ANOVA; **$p<0.01$.

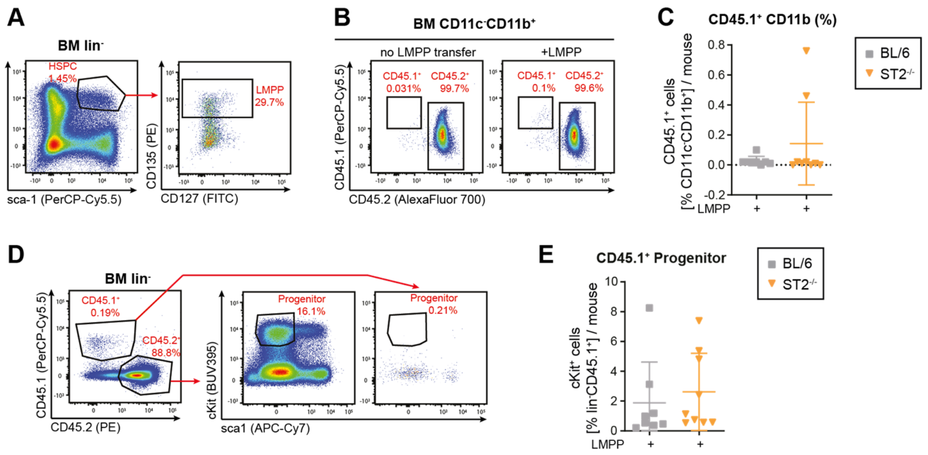

Figure S2. Transfer of ST2-competent ILC2s into ST2$^{-/-}$ recipients normalizes myelopoiesis, related to Figure 2.

(A) FACS-gating strategy for BM-resident lymphoid-primed multipotent progenitors (LMPPs). **(B)** CD45.1/CD45.2 FACS-gating for BM CD11c^{-}CD11b^{+} cells in control and LMPP-transferred mice. **(C)** Frequency of CD45.1^{+} cells (originating from the transferred LMPPs) within the CD11b^{+} myeloid compartment in BL/6 (n=8) and ST2$^{-/-}$ (n=9) recipients. **(D)** FACS-gating strategy to characterize the progenitor lin^{-}CD45.1^{+} cells. **(E)** Frequencies of cKit^{+} progenitor cells within the lin^{-}CD45.1^{+} cells in BL/6 (n=8) and ST2$^{-/-}$ (n=9) recipients.

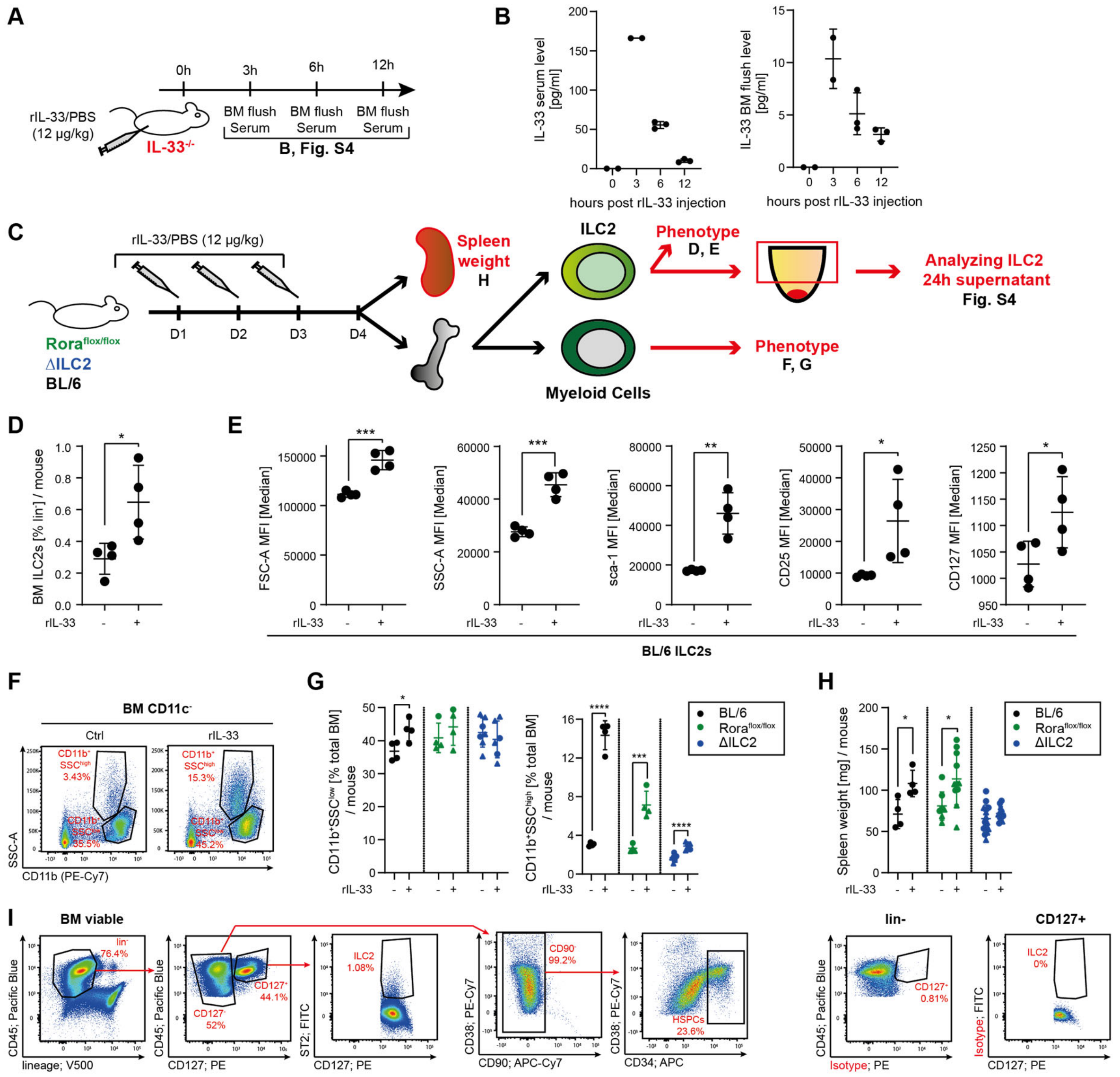

Figure S3. IL-33-activated ILC2s increase the colony-forming capacity of HSPCs and induce myeloid differentiation, related to Figure 3.

(A) Experimental layout. Recombinant (r)IL-33 was intraperitoneally injected into IL-33$^{-/-}$ mice; the mice were sacrificed after 3, 6, or 12h, and the BM fluid and serum were analyzed. **(B)** IL-33 levels measured in the serum and BM fluid of IL-33$^{-/-}$ mice injected with rIL-33 (n=2-3 per condition). **(C)** Experimental layout of the rIL-33 *in vivo* experiment. rIL-33 was injected i.p. for 3 consecutive days into BL/6, Rora$^{flox/flox}$, and ΔILC2 mice. On day 4, the mice were sacrificed, and the spleen, the BM ILC2s, and the BM myeloid cells were analyzed. **(D)** Frequency of ILC2s within the lin^{-} compartment of control and 3d rIL-33 (n=4 per group) treated BL/6 mice. **(E)** FSC, SSC, sca-1, CD25, and CD127 expression on ILC2s of control and 3d rIL-33 treated BL/6 mice (n=4 per group), measured by flow cytometry, and represented as mean fluorescence intensity (MFI). **(F)** SSC/CD11b FACS-gating for total BM CD11c^{-} cells in control and 3d rIL-33 rIL-33 treated BL/6 mice. **(G)** Frequency of CD11b^{+}SSC^{low} and CD11b^{+}SSC^{high} cells of the total BM of control and 3d rIL-33 treated BL/6 (n=4 per group), Rora$^{flox/flox}$ (n=4 per group), and ΔILC2 (n=6-7 per group) mice. **(H)** Spleen weight of control and 3d rIL-33 treated BL/6 (n=4 per group), Rora$^{flox/flox}$ (n=6-7 per group), and ΔILC2 (n=9-13 per group) mice. ● represent females and ▲ represent males in figures G-H. **(I)** FACS gating strategy for human BM-resident HSPCs and ILC2s.Statistics: two-tailed students test; *p<0.05, **p<0.01, ***p<0.001, ****p<0.0001.

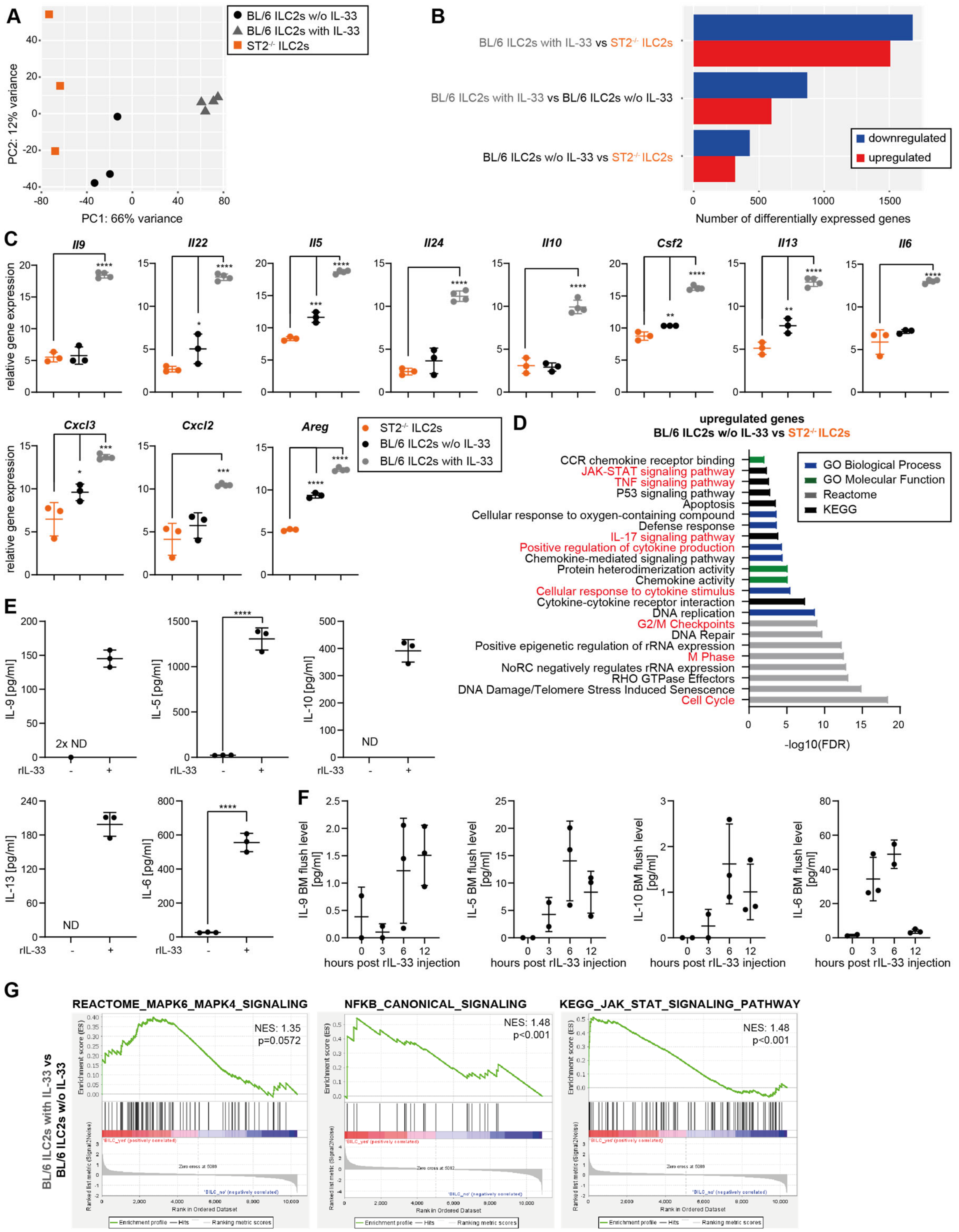

A
BL/6 ILC2s w/o IL-33
BL/6 ILC2s with IL-33
ST2-/- ILC2s
PC1: 66% variance
PC2: 12% variance
B
BL/6 ILC2s with IL-33 vs ST2-/- ILC2s
BL/6 ILC2s with IL-33 vs BL/6 ILC2s w/o IL-33
BL/6 ILC2s w/o IL-33 vs ST2-/- ILC2s
downregulated
upregulated
Number of differentially expressed genes
C
Il9
Il22
Il5
Il24
Il10
Csf2
Il13
Il6
Cxcl3
Cxcl2
Areg
relative gene expression
ST2-/- ILC2s
BL/6 ILC2s w/o IL-33
BL/6 ILC2s with IL-33
D
upregulated genes
BL/6 ILC2s w/o IL-33 vs ST2-/- ILC2s
CCR chemokine receptor binding
JAK-STAT signaling pathway
TNF signaling pathway
P53 signaling pathway
Apoptosis
Cellular response to oxygen-containing compound
Defense response
IL-17 signaling pathway
Positive regulation of cytokine production
Chemokine-mediated signaling pathway
Protein heterodimerization activity
Chemokine activity
Cellular response to cytokine stimulus
Cytokine-cytokine receptor interaction
DNA replication
G2/M Checkpoints
DNA Repair
Positive epigenetic regulation of rRNA expression
M Phase
NoRC negatively regulates rRNA expression
RHO GTPase Effectors
DNA Damage/Telomere Stress Induced Senescence
Cell Cycle
GO Biological Process
GO Molecular Function
Reactome
KEGG
-log10(FDR)
E
IL-9 [pg/ml]
2x ND
IL-5 [pg/ml]
IL-10 [pg/ml]
ND
IL-13 [pg/ml]
ND
IL-6 [pg/ml]
rIL-33
F
IL-9 BM flush level [pg/ml]
IL-5 BM flush level [pg/ml]
IL-10 BM flush level [pg/ml]
IL-6 BM flush level [pg/ml]
hours post rIL-33 injection
G
BL/6 ILC2s with IL-33 vs BL/6 ILC2s w/o IL-33
REACTOME_MAPK6_MAPK4_SIGNALING
NES: 1.35
p=0.0572
NFKB_CANONICAL_SIGNALING
NES: 1.48
p<0.001
KEGG_JAK_STAT_SIGNALING_PATHWAY
NES: 1.48
p<0.001
Enrichment score (ES)
Ranked list metric (Signal2Noise)
Rank in Ordered Dataset
Enrichment profile
Hits
Ranking metric scores

Figure S4. IL-33/ST2 signaling in ILC2 stimulates the secretion of various cytokines via the MAPK, NFκB, and JAK-STAT pathways, related to Figure 4.

(A) Principal component analysis (PCA), comparing BL/6 ILC2s cultured for 24h without or with rmIL-33 (BL/6 ILC2s w/o IL-33, BL/6 ILC2s with IL-33, respectively), and $ST2^{-/-}$ ILC2s cultured for 24h without rmIL-33 ($ST2^{-/-}$ ILC2s). **(B)** Bar plot representing the numbers of differentially expressed genes (DEGs), comparing BL/6 ILC2s w/o IL-33, BL/6 ILC2s with IL-33, and $ST2^{-/-}$ ILC2s. **(C)** Relative gene expression levels of *Il9*, *Il22*, *Il5*, *Il24*, *Il10*, *Csf2*, *Il13*, *Il6*, *Cxcl3*, *Cxcl2*, and *Areg*, measured in $ST2^{-/-}$ ILC2s, BL/6 ILC2s w/o IL-33, and BL/6 ILC2s with IL-33 by RNAseq. **(D)** Gene ontology (GO) analysis of upregulated genes (BL/6 ILC2s w/o IL-33 vs. $ST2^{-/-}$ ILC2s). A GO enrichment score of ≥3 indicates significant changes in gene expression. **(E)** IL-9, IL-5, IL-10, IL-13, and IL-6 levels, measured in the 24h supernatant of FACS-sorted BL/6 ILC2s from control mice or animals treated for 3 days with rIL-33, cultured in the absence of rmIL-33 (n=3 per group). **(F)** IL-9, IL-5, IL-10, and IL-6 levels, measured in the BM fluid of $IL\text{-}33^{-/-}$ mice treated with rIL-33 (n=2-3 per condition). **(G)** Gene set enrichment analysis (GSEA), representing the enrichment score of different gene sets, comparing BL/6 ILC2s with IL-33 with BL/6 ILC2s w/o IL-33. Statistics: two-tailed students test (E), one-way ANOVA (C); **p<0.01, ***p<0.001, ****p<0.0001.

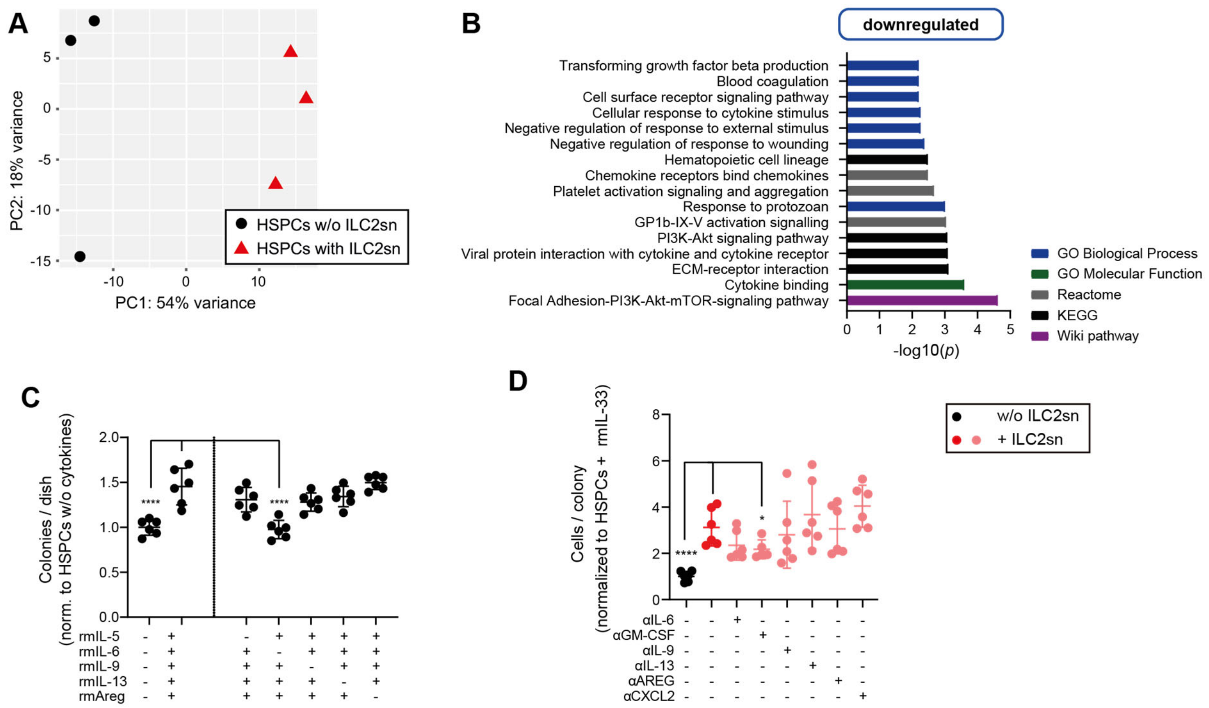

Figure S5. IL-6, GM-CSF and AREG secreted by ILC2s induce proliferation and differentiation of HSPCs, related to Figure 5.

(A) Principal component analysis, comparing BL/6 HSPCs cultured for 24h without or with ILC2 supernatant (HSPCs w/o ILC2sn, HSPCs with ILC2sn). **(B)** Gene ontology (GO) analysis of downregulated genes (HSPCs with ILC2sn vs. HSPCs w/o ILC2sn). A GO enrichment score of ≥3 indicates significant changes in gene expression. **(C)** Colony forming assay performed with $1x10^3$ FACS-purified HSPCs. Before plating in methylcellulose, HSPCs were cultured O.N. in artificial ILC2sn, consisting of rmIL-5, rmIL-6, rmIL-9, rmIL-13, and rmAREG. Single components of the artificial ILC2sn were omitted per condition. Colonies were counted 7 days post-seeding (n=6 per condition). **(D)** Colony forming assay performed with $1x10^3$ FACS-purified HSPCs. Before plating in methylcellulose, HSPCs were cultured O.N. with rmIL-33, or BL/6 ILC2sn, supplemented with different cytokine/chemokine neutralizing antibodies (αIL-6, αGM-CSF, αIL-9, αIL-13, αAREG, αCXCL2 (n=6 per condition). Statistics: one-way ANOVA; *p<0.05, **p<0.01, ***p<0.001, ****p<0.0001.

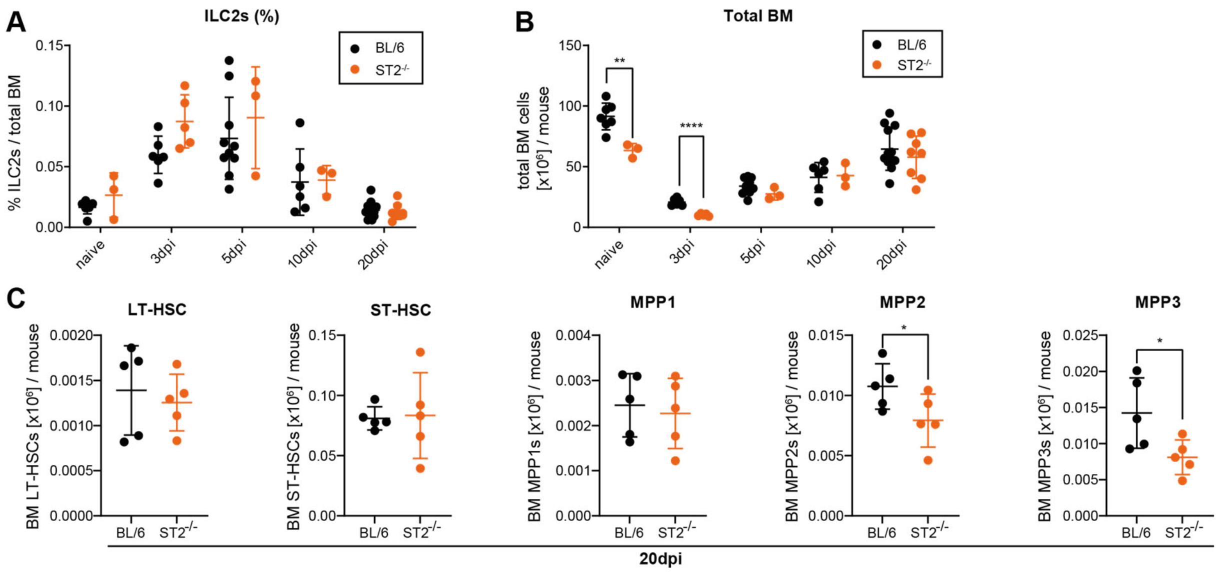

Figure S6. IL-33/ST2 signaling and ILC2s induce the expansion of HSPCs post-irradiation, related to Figure 6.
(A) BM ILC2s in naïve and sub-lethally irradiated BL/6 and $ST2^{-/-}$ mice, displayed as the percentage of total BM cells (n=3-10 per condition; pooled from 2 independent experiments). **(B)** Total BM counts in naïve and sub-lethally irradiated BL/6 and $ST2^{-/-}$ mice (n=3-10 per condition; pooled from 2 independent experiments). **(C)** LT-HSC, ST-HSC, MPP1, MPP2, and MPP2 BM counts measured in BL/6 and $ST2^{-/-}$ mice (20dpi; n=5 per group). Statistics: two-tailed Students t-test; *p<0.05, **p<0.01, ****p<0.0001.

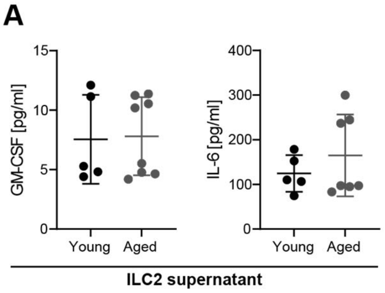

Figure S7. IL-33/ST2 signaling drives the expansion of functionally impaired and myeloid skewed HSPCs during aging, related to Figure 7.
(A) GM-CSF and IL-6 levels were measured in the 24h supernatant of FACS-purified ILC2s from young and aged BL/6 mice.

Supplementary Tables

Table S1. Clinical characteristics of human samples enrolled in the study.

Sample ID	Sex	Age (year)	Race	Ethnicity
**Sample #1**	NA	NA	NA	NA
**Sample #2**	M	68	NA	NA
**Sample #3**	F	52	NA	NA
**Sample #4**	F	89	NA	NA
**Sample #5**	F	77	NA	NA

NA: not available.
